# Stress granules sequester Alzheimer’s disease-associated gene transcripts and regulate disease-related neuronal proteostasis

**DOI:** 10.18632/aging.204737

**Published:** 2023-05-22

**Authors:** Kaoru Sato, Ken-ichi Takayama, Satoshi Inoue

**Affiliations:** 1 Systems Aging Science and Medicine, Tokyo Metropolitan Institute for Geriatrics and Gerontology (TMIG), Itabashi-ku, Tokyo 173-0015, Japan; 2 Integrated Research Initiative for Living Well with Dementia (IRIDE), TMIG, Itabashi-ku, Tokyo 173-0015, Japan

**Keywords:** stress granule, RNP granule, eCLIP-seq, G3BP, Alzheimer’s disease

## Abstract

Environmental and physiological stresses can accelerate Alzheimer’s disease (AD) pathogenesis. Under stress, a cytoplasmic membraneless structure termed a stress granule (SG) is formed and is associated with various neurodegenerative disorders, including AD. SGs contain translationally arrested mRNAs, suggesting that impaired RNA metabolism in neurons causes AD progression; however, the underlying mechanism remains unclear. Here, we identified numerous mRNAs and long non-coding RNAs that are directly targeted by the SG core proteins G3BP1 and G3BP2. They redundantly target RNAs before and after stress conditions. We further identified RNAs within SGs, wherein AD-associated gene transcripts accumulated, suggesting that SGs can directly regulate AD development. Furthermore, gene-network analysis revealed a possible link between the sequestration of RNAs by SGs and the impairment of protein neurohomeostasis in AD brains. Together, our study provides a comprehensive RNA regulatory mechanism involving SGs, which could be targeted therapeutically to slow AD progression mediated by SGs.

## INTRODUCTION

Alzheimer’s disease (AD) is a neurodegenerative disorder characterized by neuronal loss and learning/memory deficits. In the brains of AD patients, deposits of amyloid-β (Aβ) plaques and neurofibrillary tangles (NFTs) of hyperphosphorylated tau proteins have been observed [1–3]. Aβ is generated from the amyloid precursor protein (APP) through sequential proteolytic cleavage involving β-site APP-cleaving enzyme 1 (BACE1) and γ-secretase [1, 2]. Several studies indicate that environmental and physiological stress can accelerate AD pathogenesis [4–7]. In response to stress exposure, RNA-containing non-membrane foci, termed stress granules (SGs), are formed in the cytoplasm by liquid–liquid phase separation (LLPS) [8–14]. Hundreds of proteins, mainly RNA-binding proteins (RBPs), are localized to SGs, including the Ras-GTPase-activating protein SH3-domain-binding protein (G3BP) family, the core protein of SGs [6, 9, 11, 15–19]. Humans possess two *G3BP* genes, *G3BP1* and *G3BP2*, which are evolutionarily conserved across species [11, 19]. G3BP proteins are RBPs, as indicated by the presence of an RNA recognition motif (RRM), in addition to a nuclear transport factor 2 (NTF2) domain and two intrinsically disordered regions (IDRs) [19]. IDRs and RRM cooperate to modulate LLPS and contribute to SG assembly [10–12]. G3bp1 plays an essential role in neuronal function, as demonstrated in a mouse model, where genetic deletion of G3bp1 causes late embryonic lethality with severe neuronal cell death in the brain [20]. Furthermore, *G3bp1*-deficient mice exhibit aberrant synaptic plasticity and calcium homeostasis in the hippocampus, which is linked to neurocognitive and neurodegenerative disorders associated with aging in humans [21–23]. Testis-specific *G3bp2* deficiency leads to increased apoptosis in the testes, suggesting its crucial role in maintaining tissue homeostasis [24].

Generally, SGs are transient structures that protect cells, including neurons, from harmful stresses by globally arresting mRNA translation and store various proteins and RNAs [9, 25, 26]. Meanwhile, chronic stress causes the deposition of persistent SGs, which are further converted to pathological SGs that exhibit an aberrant solid-like state [13, 15]. Long-term SGs are associated with neurodegenerative diseases and appear to function as a nidus for the aggregation of pathological proteins, including phosphorylated tau inclusions in neurons [13, 27–29]. The strong depositions of G3BP and other protein components of SGs, including T-cell intracellular antigen-1 (TIA-1) and tristetraprolin (TTP), have been observed in the brains of human AD patients and AD mouse models [30–33]. In addition to the foci of the aggregation of disease-associated proteins, numerous RNAs are also accumulated in SGs, wherein the translation of most mRNAs is stalled during times of cellular stress. This suggests that, in AD brains, pathological SGs can sequester those RNAs irreversibly, causing the collapse of overall RNA metabolism in neurons, subsequently leading to the loss of neuronal cells and cognitive disorders [8, 13, 27]. However, the molecular mechanisms underlying SG-mediated RNA regulation remain unclear.

In this study, we conducted a genome-wide investigation of the G3BP1- and G3BP2-bound RNAs using enhanced cross-linking and immunoprecipitation-sequencing (eCLIP-seq) in the human neuroblastoma (NB) cell line SH-SY5Y. Both proteins bound to numerous mRNAs and long non-coding RNAs (lncRNAs). Furthermore, examination of SG-enriched RNAs revealed that most of them overlapped with G3BP1/2-bound RNAs, suggesting their direct contribution to SG RNA assembly. Additionally, SG-enriched RNAs contained multiple gene transcripts associated with AD, herein referred to as AD-associated genes, which are longer, AT-rich transcripts. Moreover, gene-network analysis using SG-enriched genes and protein co-expression data revealed a possible link between the accumulation of RNAs in SGs and consequent changes in a set of protein levels in AD brains. These findings provide a comprehensive RNA regulatory mechanism involving SGs, which could be targeted therapeutically to slow AD progression mediated by SGs.

## RESULTS

### G3BP1 and G3BP2 interact with numerous mRNAs and lncRNAs

**Figure 1:**
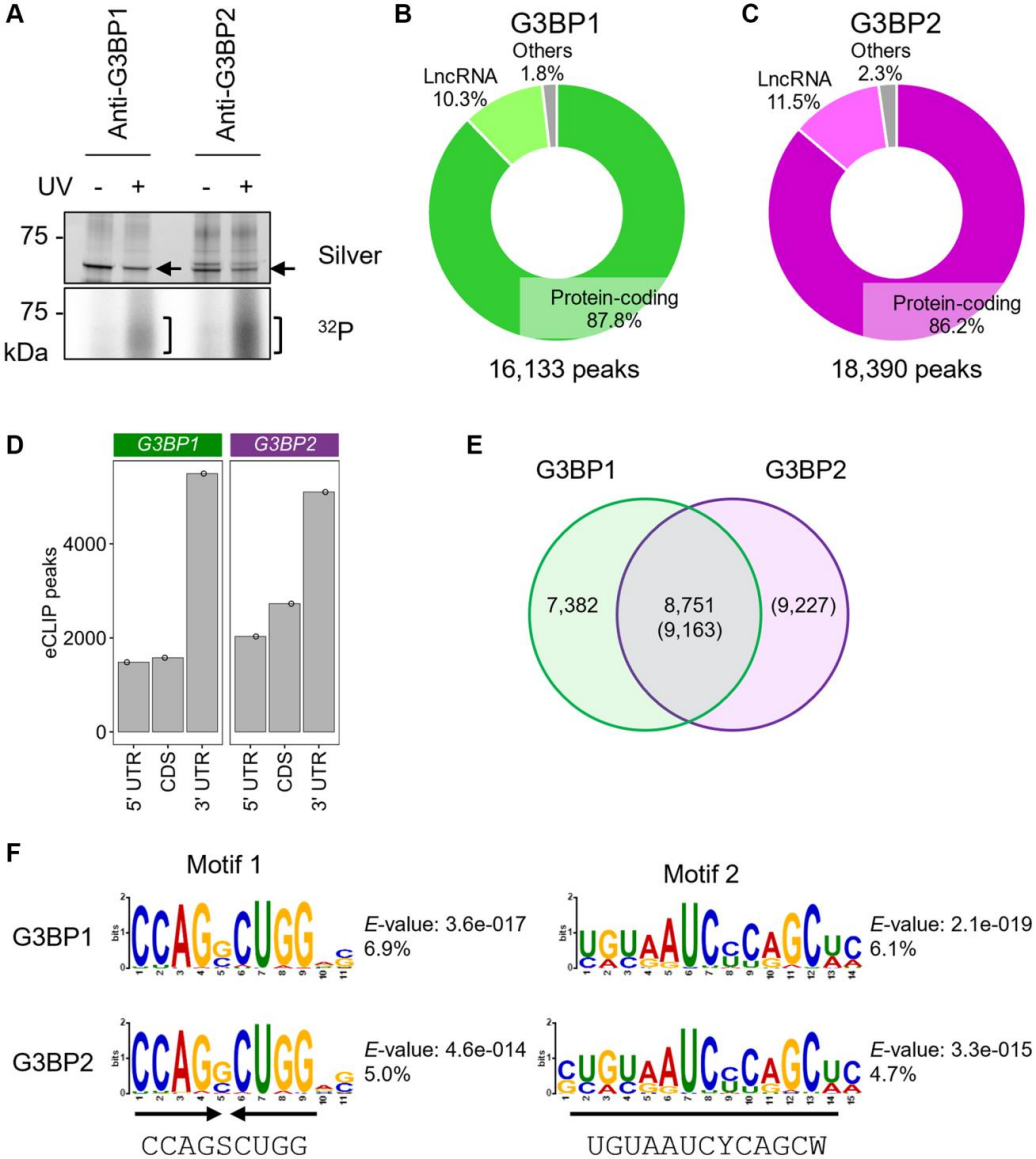
**eCLIP-seq of G3BP1 and G3BP2 in SH-SY5Y cells under normal conditions.** (**A**) Silver staining of immunopurified G3BP1 and G3BP2, and ^32^P autoradiograph in SH-SY5Y cells with and without UV crosslinking. Arrows and square brackets indicate immunopurified proteins and radioactively labelled RNAs, respectively. (**B**, **C**) Pie chart depicting the relative contribution of gene categories for G3BP1- (**B**) and G3BP2-bound RNAs (**C**). (**D**) Bar plot showing G3BP1 and G3BP2 preferentially associates with 3′UTR of the target mRNA. (**E**) Venn diagram depicting the overlapped eCLIP-peaks between G3BP1- and G3BP2-bound RNAs. (**F**) Motifs enriched within eCLIP-peaks of G3BP1- and G3BP2-bound RNAs.

Both G3BP1 and G3BP2 contain an RRM domain, that is an RNA-binding motif found in many RNA-binding proteins, with a variety of RNA-binding preferences [19]. However, it remains unknown which RNA types are captured by these proteins in human neuronal cells. Cross-linking immunoprecipitation (CLIP) experiments showed that endogenous G3BP1 and G3BP2 were efficiently cross-linked with RNAs upon UV irradiation in SH-SY5Y cells (Figure 1A, Supplementary Figure 1A). To identify the binding RNAs, eCLIP-seq was performed in SH-SY5Y cells. We performed the experiments in duplicate and used common peaks for analyses. We identified 16,133 and 18,390 peaks for G3BP1 and G3BP2 RNA binding sites, respectively, of which approximately 86% were localized to transcripts for protein-coding genes, namely mRNAs, and 10–12% for lncRNAs (Figure 1B, 1C) based on the GENCODE gene annotation [34]. Out of 19,942 protein-coding genes, 4,548 and 5,009 had at least one peak of G3BP1 and G3BP2, respectively, which was enriched in the 3′UTR region, followed by the coding sequence (CDS) and 5′UTR regions (Figure 1D). A total of 3,365 protein-coding genes overlapped (Supplementary Figure 1B). We confirmed that G3BP proteins interact with RNAs which possess their eCLIP-peaks, such as *spectrin beta, non-erythrocytic 1* (*SPTBN1*), *protein kinase C alpha* (*PRKCA*), and *transcription factor 4* (*TCF4*), by RNA immunoprecipitation (RIP), whereas two genes without eCLIP-peaks, *macrophage migration inhibitory factor* (*MIF*) and *heme oxygenase 2* (*HMOX2*), were less abundant (Supplementary Figure 1C). Moreover, their interactions were also observed in other human NB cell lines, NB9 and NB69 (Supplementary Figure 1D, 1E), suggesting that RNAs identified in the eCLIP experiments are targeted by G3BP proteins in other NB cell lines. Approximately 33% of peaks were shared between both proteins (Figure 1E), and similar RNA-binding motifs were enriched within the peaks (Figure 1F). The most predominant RNA-binding motif of G3BP1 was a palindromic RNA sequence, in which the consensus sequence was CCAGSCUGG (S indicates G/C) (Figure 1F). This motif was also found in G3BP2. The palindromic sequence forms a hairpin structure, as predicted by CentroidFold, a prediction tool for RNA secondary structures (Supplementary Figure 1F) [35], suggesting that G3BP1 and G3BP2 may recognize higher-order RNA structures. The second RNA-binding motif commonly found in both proteins, UGUAAUCYCAGCW (Y and D indicate C/U and G/A/U, respectively), was enriched in approximately 5% of the peaks (Figure 1F). Taken together, G3BP1 and G3BP2 primarily target overlapping transcripts by recognizing similar RNA sequences or secondary structures in SH-SY5Y cells.

### G3BP-bound RNA repertoires slightly changed after stress

**Figure 2:**
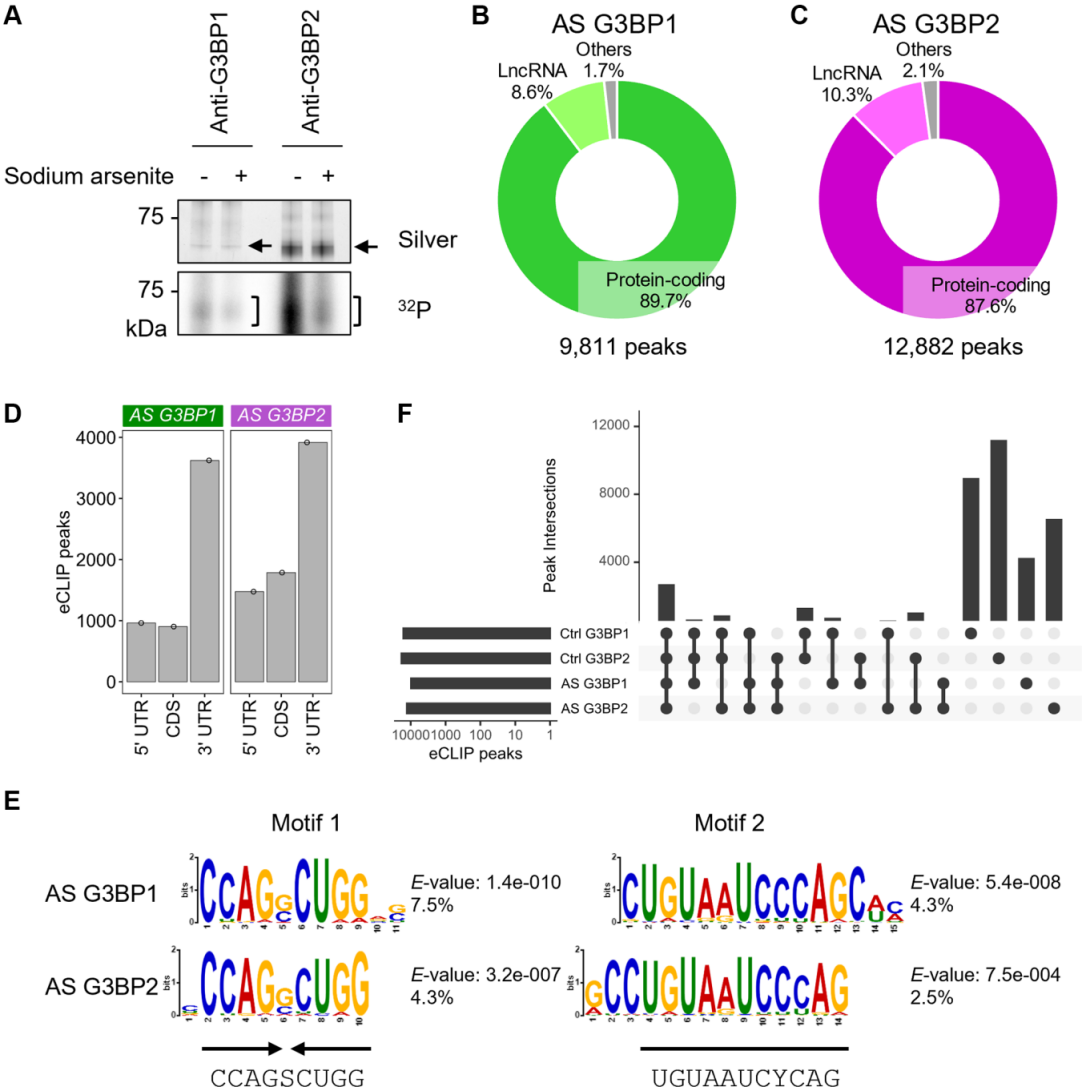
**eCLIP-seq of G3BP1 and G3BP2 in sodium arsenite (AS)-treated SH-SY5Y cells.** (**A**) Silver staining of immunopurified G3BP1 and G3BP2 and ^32^P autoradiogram in SH-SY5Y cells before and after AS treatment. n.i.: non-immune IgG used as an IP negative control. Arrows and square brackets indicate immunopurified proteins and radioactively labelled RNAs, respectively. (**B**, **C**) Pie chart depicting the relative contribution of gene categories for G3BP1- (**B**) and G3BP2-bound RNAs (**C**). (**D**) Bar plot showing G3BP1 and G3BP2 preferentially associates with 3′UTR of the target mRNA. (**E**) Motifs enriched within eCLIP-peaks of G3BP1- and G3BP2-bound RNAs. (**F**) Intersection of eCLIP-peaks across G3BP1- and G3BP2-bound RNAs before (Control, Ctrl) and after AS treatment.

G3BP1 and G3BP2 are core protein components of SGs and indeed formed SGs in the cytoplasm of SH-SY5Y cells under stress induced by sodium arsenite (AS) (Supplementary Figure 1G, 1H). CLIP experiments revealed that, despite slight signal reductions, G3BP1 and G3BP2 still bind RNAs under stress conditions (Figure 2A). To identify global RNA-binding changes for G3BP1 and G3BP2 under stress, we performed eCLIP-seq analyses using AS-treated SH-SY5Y cells. Similar to the untreated cells, we identified 9,811 and 12,882 peaks for G3BP1 and G3BP2, respectively, of which over 80% were annotated to transcripts for protein-coding genes and 8–10% for lncRNAs (Figure 2B, 2C). These peaks were enriched in the 3′UTR regions, followed by the CDS and 5′UTR regions of protein-coding genes (Figure 2D). The interactions between G3BP proteins and *SPTBN1*, *PRKCA*, and *TCF4* mRNAs were also confirmed by RIP in SH-SY5Y as well as NB9 and NB69 cells (Supplementary Figure 1I–1K). Motifs similar to the palindromic and the second motif, which are prominent for G3BP1- and G3BP2-bound RNAs in untreated cells, were also found most abundantly in AS-treated cells (Figure 2E). A previous study showed a possible RNA-binding motif, including GGAU, for G3BP2 *in vitro* [36]. A similar motif was identified but to a lesser extent (Supplementary Figure 1L), indicating that both G3BP1 and G3BP2 proteins may rarely bind to RNAs through the known G3BP2-binding motif in SH-SY5Y cells. Almost all protein-coding genes that possessed G3BP1 and G3BP2 eCLIP-peaks overlapped (Supplementary Figure 1M). Around 20% of the peaks were shared between all four samples (Figure 2F). Although the changes in the RNA binding profiles for G3BP1 and G3BP2 were modest, these observations suggest that even under normal conditions, both proteins bind to their target RNAs to be ready for sudden stress to protect these RNAs.

### Identification of transcripts in the SG dense cores

**Figure 3:**
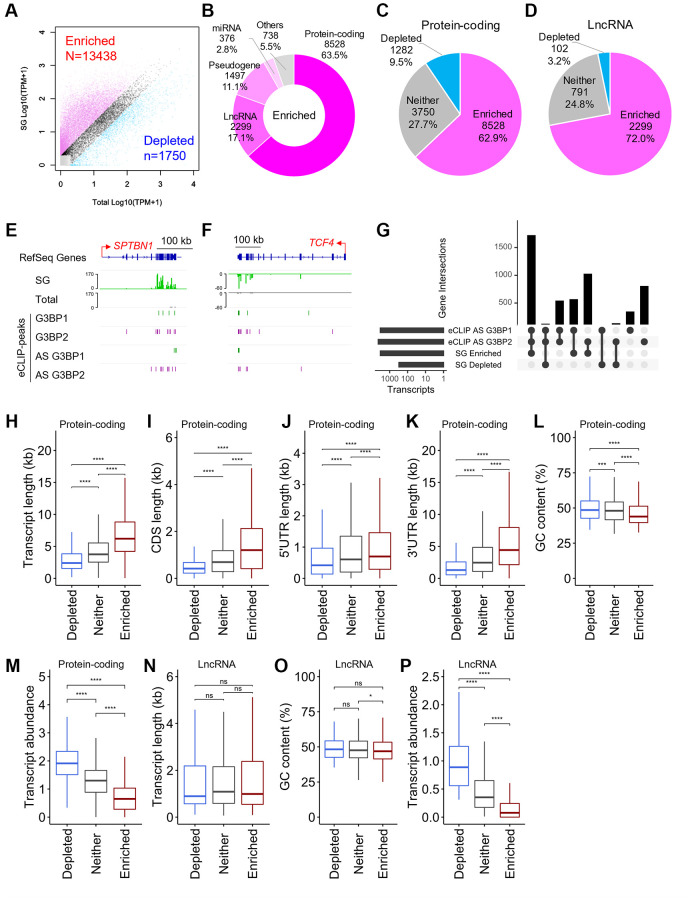
**Physical basis of RNAs recruited to SGs.** (**A**) Scatter plot depicting RNA abundance in SG purified RNAs and “Total” RNAs. Red dots indicate RNAs that are enriched (Fold change >2 and TPM of SG >1) in SG purified RNA compared to Total RNA. Blue dots indicate RNAs that are depleted (Fold change <0.5 and TPM of Total >1) in SG purified RNA compared to Total RNA. Dark gray dots indicate RNAs that are either not enriched or fail to meet the fold change requirement, referred to as “Neither.” Light gray dots indicate RNAs with TPM <1. (**B**) Pie chart depicting the relative contribution of gene categories for SG-enriched RNAs. (**C**, **D**) Pie chart depicting the relative contribution of each class of RNA (SG-enriched, SG-depleted, or “Neither”) for protein-coding genes (**C**) and lncRNAs (**D**), respectively. (**E**, **F**) Density profiles of normalized reads from SG RNA-seq (indicated in SG and Total) and eCLIP-seq peaks on *SPTBN1* (**E**) and *TCF4* (**F**). (**G**) Intersection across eCLIP-peaks of G3BP1- and G3BP2-bound RNAs, and SG-enriched and SG-depleted RNAs in AS treated SH-SY5Y cells. (**H**–**P**) Boxplots depicting total transcript length (**H** and **N**), CDS length (**I**), 5′UTR length (**J**), 3′UTR length (**K**), GC content (**L** and **O**), and abundance of RNA (**M** and **P**) of (**H**–**M**) protein-coding genes and (**N**–**P**) long non-coding RNAs (lncRNAs), respectively, for each of the three classes of mRNA localization during stress: SG enriched mRNAs, SG depleted mRNAs, or ”Neither”. Statistical significances were assessed by Wilcoxon rank sum test. ns: not significant, ^*^p < 0.05, ^***^p < 0.001, ^****^p < 0.0001.

SGs have dense cores, which are a potential source of insoluble protein-RNA aggregates [6, 17, 18]. Both G3BP proteins were efficiently detected in the soluble fraction, which was subjected to CLIP experiments in this study, in untreated SH-SY5Y cells, whereas considerable amounts of proteins were found in the insoluble fraction under stress conditions (Supplementary Figure 2A). To identify RNAs within the SG cores in SH-SY5Y cells, we purified the cores by immunoprecipitation (IP) with an anti-G3BP1 antibody according to a previously reported procedure, in which human osteosarcoma U2OS cells were used [37]. We first confirmed that the procedure was well adapted to SH-SY5Y cells, and detected multiple proteins including both G3BP proteins in the SG cores (Supplementary Figure 2B–2D). Next, we purified RNAs from SG cores from SH-SY5Y cells. The SG cores and lysate containing the total RNA population, referred to as SG and Total, respectively, were subjected to RNA-seq analysis. Out of 60,612, 13,438 transcripts in the GENCODE gene annotation [34] were enriched in the SG cores (>2-fold change, >1 transcripts per million (TPM) in SG), whereas 1,750 transcripts were depleted in the SG cores (<0.5-fold change, >1 TPM in Total) (Figure 3A). RNAs with very low levels were excluded from subsequent analysis, and the remaining transcripts, partitioned similarly between the SG cores and total RNA, were referred to as “Neither”. Out of 13,438 SG-enriched RNAs, 8,528 were assigned to protein-coding genes, followed by lncRNAs, which accounted for 17.1% (Figure 3B). Sixty to seventy percent of the transcripts for mRNAs and lncRNAs were enriched in the SG core, whereas less than 10% were depleted (Figure 3C, 3D), indicating that most mRNAs and lncRNAs were partitioned into the SG cores. Out of 13,438, 3,323 (24.7%) SG-enriched RNAs, including *SPTBN1* and *TCF4* (Figure 3E, 3F), possessed at least one eCLIP-peak for G3BP1 and G3BP2 under stress conditions (Figure 3G). We confirmed the enrichment of these RNAs in SGs using reverse transcription-quantitative PCR (RT-qPCR) against SG-RNAs and found that SG-enriched RNAs, including *SPTBN1*, *TCF4*, and *PRKCA*, were enriched in SGs in SH-SY5Y as well as NB9 and BN69 cells, whereas SG-depleted RNAs, such as *emopamil-binding protein-like* (*EBPL*), *MIF* and *tumor protein, translationally-controlled 1* (*TPT1*), were not abundant (Supplementary Figure 2E–2H). These observations indicate that G3BP1 and G3BP2 play major roles in the loading of RNAs into SGs in human neuronal cells. The remaining SG-enriched RNAs lacked G3BP1 and G3BP2 binding peaks, suggesting that they could be recruited to SGs in a G3BP-independent manner. This is because SGs consist of multiple RBPs, such as TIA-1 and TPP [9], which can recruit target RNAs to SGs through direct interactions. Furthermore, the existence of subtypes of SGs with heterogeneous protein and RNA content has recently been suggested [38].

### SG-enriched RNAs are long and AT-rich

In U2OS cells, SG-enriched mRNAs were shown to be longer, have a lower GC content and lower abundance than SG-depleted RNAs [37]. Similar to U2OS cells, SG-enriched mRNAs were much longer than SG-depleted and “Neither” ones in SH-SY5Y cells (Figure 3H). We further explored the different contributions of the 5′UTR, CDS, and 3′UTR regions. We found that the length of each region was different between enriched and depleted RNAs in SGs (Figure 3I–3K), indicating that these lengths are also important metrics for determining SG accumulation in SH-SY5Y cells. It has been shown that transcripts with longer 5′UTRs and lower translation efficiency tend to be enriched in SGs. Longer 5′UTR lengths can decrease translation initiation due to RNA structure and competitive binding by small non-coding RNAs such as microRNAs [39–41]. In further support of this, we observed that RNAs preferentially localized to SGs have lower GC contents, are AT-rich, and are less abundant in SH-SY5Y cells (Figure 3L, 3M), which are general features observed for poorly translated mRNAs [42]. In contrast, lncRNAs showed no difference in transcript length between enriched and depleted RNAs in SGs (Figure 3N), indicating that this cannot be a significant predictor of RNA accumulation in SGs in SH-SY5Y cells. SG-enriched lncRNAs showed slightly lower GC content than “Neither” RNAs (Figure 3O). Additionally, similar to mRNAs, SG-enriched lncRNAs were less abundant than SG-depleted and “Neither” ones (Figure 3P). These observations suggest that AT-rich lncRNAs with low abundance tend to accumulate in SGs in SH-SY5Y cells.

### Distinct roles of G3BP1 and G3BP2 in partitioning of RNAs to SGs

**Figure 4:**
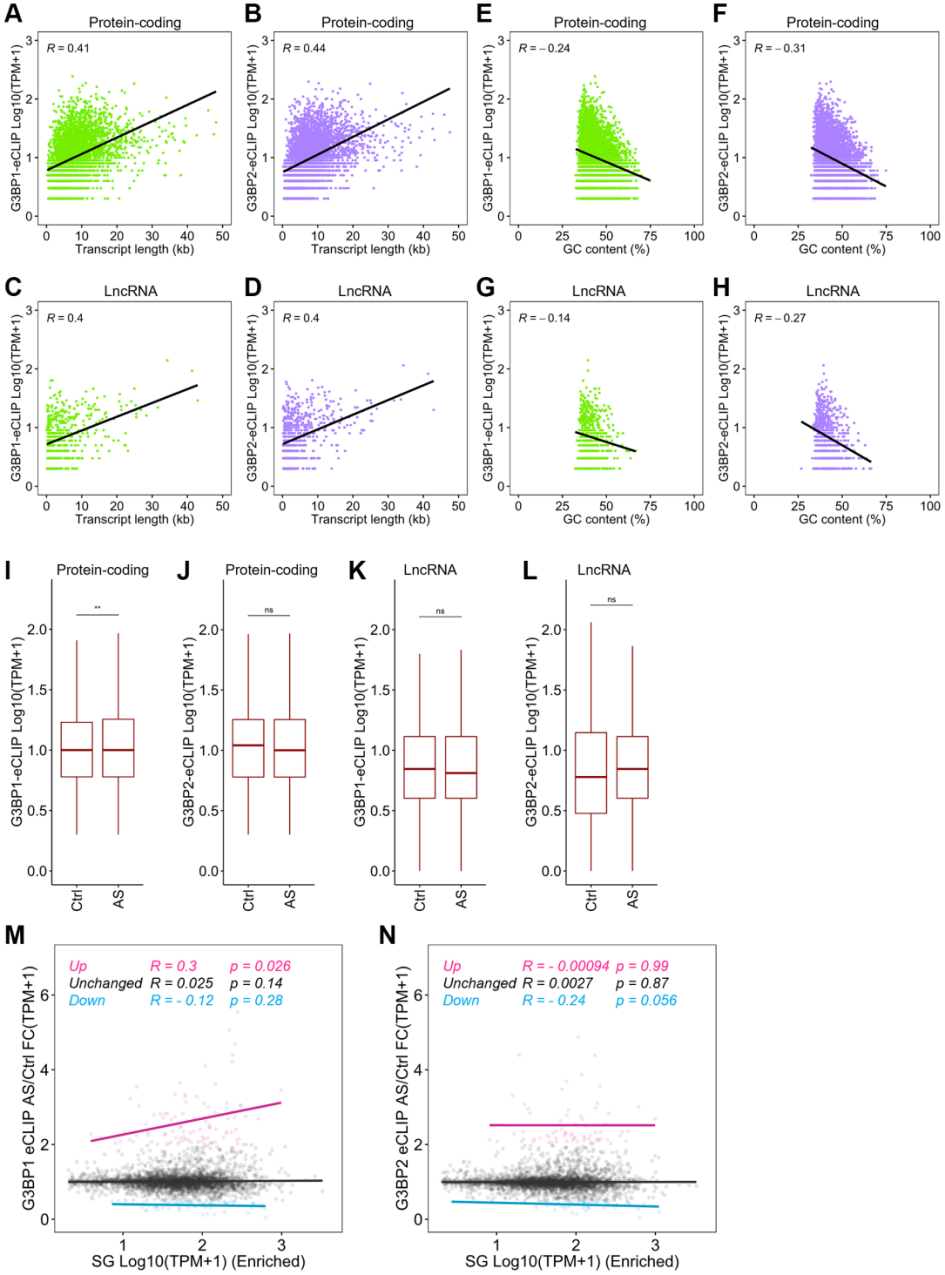
**Distinct RNA targeting of G3BP1 and G3BP2 after stress.** (**A**, **B**) Scatter plot depicting the correlation between mRNA length and the RNA binding levels of G3BP1 (**A**) and G3BP2 (**B**) with eCLIP-peaks. R indicates the Pearson correlation coefficient. (**C**, **D**) Scatter plot depicting the correlation between GC content of mRNAs and the RNA binding levels of G3BP1 (**C**) and G3BP2 (**D**) with eCLIP-peaks. (**E**, **F**) Scatter plot depicting the correlation between lncRNA length and the RNA binding levels of G3BP1 (**E**) and G3BP2 (**F**) with eCLIP-peaks. (**G**, **H**) Scatter plot depicting the correlation between GC content of lncRNAs and the RNA binding levels of G3BP1 (**G**) and G3BP2 (**H**) with eCLIP-peaks. (**I**–**L**) Boxplots depicting the G3BP1- and G3BP2-bound mRNA and lncRNA levels of SG-enriched RNAs before and after AS treatment. Statistical significance was computed with Wilcoxon rank sum test. ns: not significant, ^**^p < 0.01. (**M**, **N**) Scatter plot showing the correlation between SG enriched RNA levels and change in G3BP1- and G3BP2-bound RNA levels upon AS treatment. R and *p* indicate Pearson correlation coefficient and *p*-value, respectively.

We showed above that longer and more AT-rich transcripts tend to assemble into SGs. We further found that the length of mRNAs and lncRNAs positively correlated with the number of eCLIP-peaks for G3BP1 and G3BP2 in each gene (Figure 4A–4D), while GC content was negatively correlated with the peaks of mRNAs and lncRNAs (Figure 4E–4H). These results suggest that G3BP1 and G3BP2 likely contribute to the assembly of both mRNAs and lncRNAs to SGs through direct interaction. To further investigate this, we examined the changes in the RNA-binding levels of G3BP1 and G3BP2 before and after AS treatment in SH-SY5Y cells. G3BP1, but not G3BP2, binding levels on SG-enriched mRNAs were slightly but significantly increased under stress (Figure 4I, 4J, compare control (Ctrl) enriched with AS enriched). LncRNAs also showed marginal changes, but did not reach statistical significance (Figure 4K, 4L). We extended the analysis to changes in RNA binding levels for G3BP1 and G3BP2 in SG-enriched mRNAs after stress stimuli. G3BP1-bound mRNA levels increased after AS treatment and were slightly but significantly positively correlated with those of SG-enriched mRNAs (Figure 4M). In contrast, reduced RNA-binding levels for G3BP2 showed a slight but significant decrease in RNA levels enriched in SGs (Figure 4N). These observations suggest that stress induces an increase in G3BP1 binding to mRNAs, promoting their enrichment in SGs, while the mRNA binding of G3BP2 was mitigated by stress, which elicited accumulation of G3BP2-free mRNAs to SGs.

### Long mRNAs and lncRNAs are associated with AD

**Figure 5:**
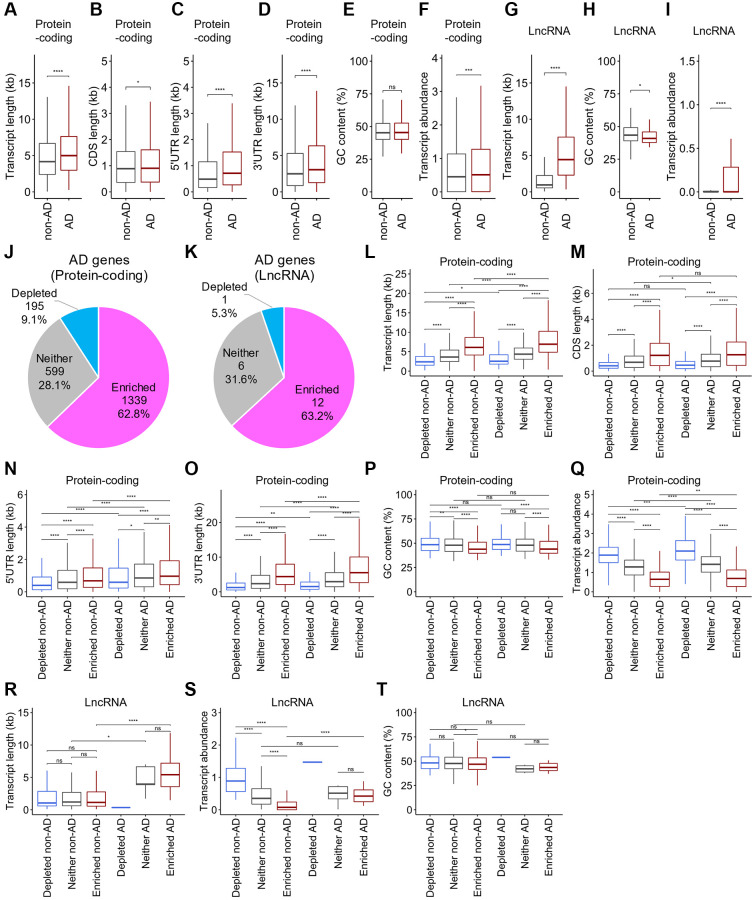
**Distinct distribution of the physical basis for AD-associated RNAs after stress.** (**A**–**J**) Boxplots depicting total transcript length (**A** and **G**), CDS length (**B**), 5′UTR length (**C**), 3′UTR length (**D**), GC content (**E** and **H**), and abundance (**F** and **I**) of mRNAs (**A**–**F**) and lncRNAs (**G**–**I**), respectively, for each of AD-associated RNAs (AD) and other RNAs (non-AD). Statistical significances were assessed by Wilcoxon rank sum test. ns: not significant, ^*^p < 0.05, ^**^p < 0.01, ^***^p < 0.001, ^****^p < 0.0001. (**J**, **K**) Pie chart depicting the relative contribution of each class of RNA (SG-enriched, SG-depleted, or “Neither”) for mRNAs (**J**) and lncRNAs (**K**), respectively. (**L**–**T**) Boxplots depicting total transcript length (**L** and **R**), CDS length (M), 5′UTR length (**N**), 3′UTR length (**O**), GC content (**P** and **S**), and abundance (**Q** and **T**) of mRNAs (**L**–**Q**) and lncRNAs (**R**–**T**), respectively, for each of AD-associated RNAs (AD) and other RNAs (non-AD).

Sequestration of gene transcripts associated with AD pathology by SGs in neurons leads to AD progression. However, whether SGs sequester these transcripts has not been examined. To identify transcripts associated with AD pathology regulated by SGs in neuronal cells, we obtained a list of genes associated with AD from DisGeNET, a database of gene-disease associations [43], referred to as AD-associated genes, wherein 3,096 protein-coding genes and 52 lncRNAs were included. Remarkably, we found that the transcripts for AD-associated protein-coding genes, referred to as AD-associated mRNAs (*n* = 3,096), were much longer than non-AD-associated mRNAs (*n* = 16,846) (Figure 5A). Furthermore, examination of the contributions of the 5′UTR, CDS, or 3′UTR regions revealed that the lengths of each region were significantly different between AD-associated and non-AD-associated mRNAs (Figure 5B–5D). GC content showed no significant difference between the gene groups, whereas AD-associated mRNAs were abundantly expressed (Figure 5E, 5F). Similar to mRNAs, AD-associated lncRNAs (*n* = 52) also showed longer transcript lengths than non-AD lncRNAs (*n* = 16,836) (Figure 5G). Additionally, lower GC content and higher expression levels were observed (Figure 5H, 5I). Taken together, mRNAs and lncRNAs of AD-associated genes have a propensity to possess physical features that are likely to be sequestered by SGs.

### Longer AD-associated mRNAs and lncRNAs tended to be enriched to SGs

To determine whether SGs indeed sequester transcripts for AD-associated genes, we explored the different distributions of AD-associated mRNAs and lncRNAs in SGs. Approximately 63% of mRNAs and lncRNAs for AD-associated genes were enriched in SGs in SH-SY5Y cells (Figure 5J, 5K). The ratios were relatively similar to those of total mRNAs and lncRNAs (compared to Figure 3C, 3D), suggesting that RNAs are not particularly enriched in SGs simply because they are associated with AD. Next, we examined the physical basis of AD-associated mRNAs and lncRNAs. We observed that AD-associated mRNAs enriched in SGs were significantly longer than non-associated mRNAs (Figure 5L). Notably, regardless of their association with AD, SG-enriched mRNAs exhibited typically longer CDSs and UTRs than SG-depleted and “Neither” ones. Significant contributions of the lengths of 5′UTRs and 3′UTRs but not of CDSs were observed between them (Figure 5M–5O). Additionally, GC content was lower in the SG-enriched mRNAs than in the SG-depleted and “Neither” ones, but no significant difference was seen between AD- and non-AD-associated gene transcripts (Figure 5P). AD-associated mRNAs were somewhat more abundant than non-AD-associated mRNAs, although SG-enriched mRNAs were typically less abundant in either case (Figure 5Q). Similar to AD-associated mRNAs, AD-associated lncRNAs enriched to SGs showed a longer transcript length and higher abundance than non-AD-associated ones, but no significant difference between SG-enriched AD-associated lncRNAs and “Neither” ones was observed (Figure 5R, 5S). Almost no significant difference in the GC content was observed (Figure 5T). Statistical analyses of SG-depleted AD-associated lncRNAs were not performed because they included only one gene (Figure 5R–5T). Taken together, these results indicate that both AD-associated mRNAs and lncRNAs that tend to aggregate to SGs possess significantly longer transcript lengths and relatively higher RNA levels than non-AD ones. Therefore, SG aggregability is a possible metric for distinguishing AD-associated RNAs.

### Impact on change in transcriptome upon loss of G3BP1 and G3BP2 proteins

**Figure 6:**
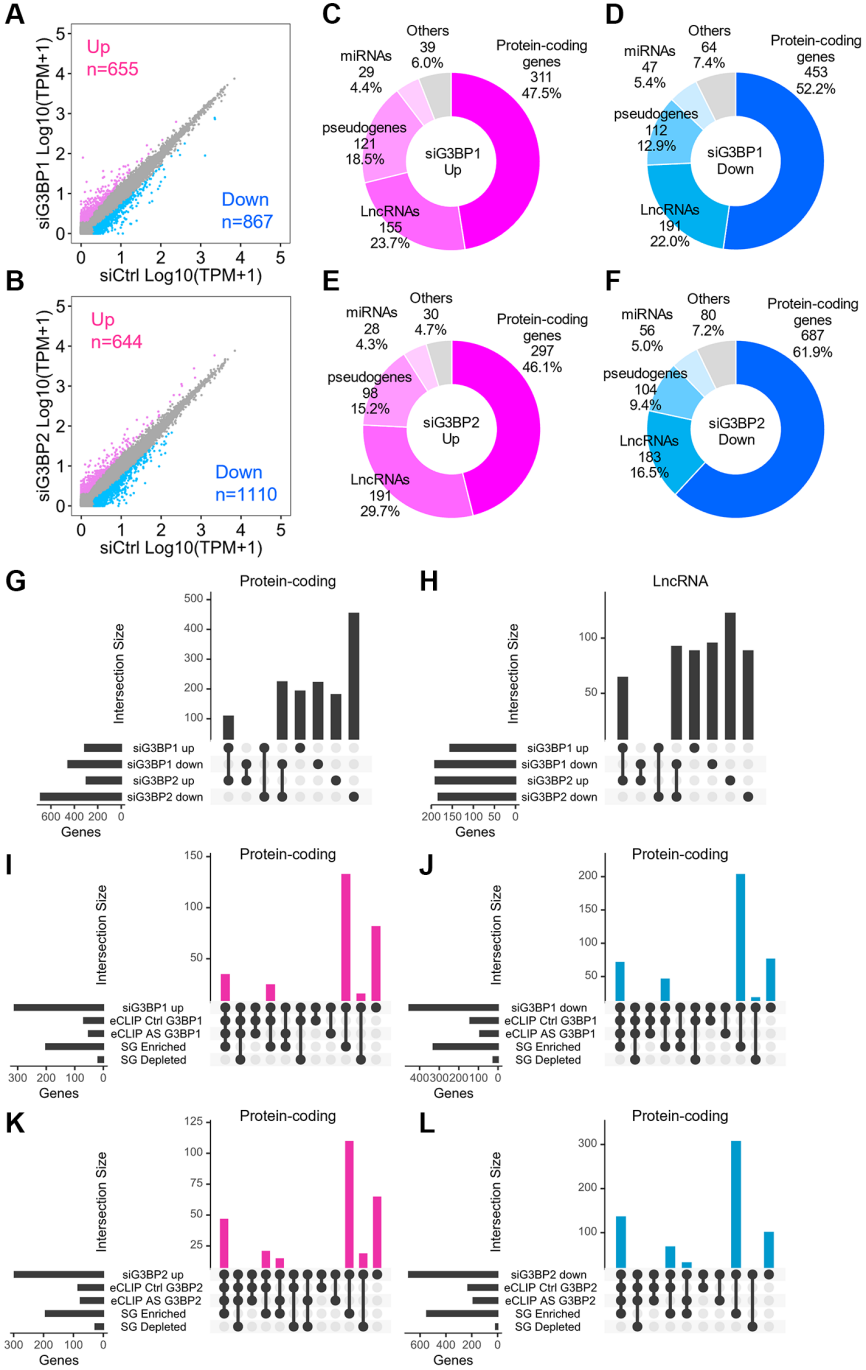
**Changes in RNA levels after the depletion of G3BP1 and G3BP2, and AS-treatment in SH-SY5Y.** (**A**, **B**) Scatter plot depicting RNA abundance in G3BP1-depleted (**A**) and G3BP2-depleted cells (**B**). Red, blue, and gray dots indicate genes upregulated, downregulated, and unchanged upon knockdown, respectively. siCtrl indicates control knockdown. (**C**–**F**) Pie charts depicting the relative contribution of gene categories for upregulated (**C** and **E**) and downregulated (**D** and **F**) genes upon depletion of G3BP1 and G3BP2, respectively. (**G** and **H**) Intersections for upregulated or downregulated genes of protein-coding genes (**G**) and lncRNAs (**H**) upon knockdown. (**I**–**L**) Intersections across upregulated, downregulated genes upon knockdown and enriched RNAs based on eCLIP-seq and SG RNA-seq of protein-coding genes.

To understand the outcomes of RNA binding of G3BP1 and G3BP2, we first investigated changes in the transcriptome upon their depletion. Global steady-state RNA levels were determined by sequencing total RNAs in G3BP1- and G3BP2-depleted SH-SY5Y cells (Figure 6A, 6B). We confirmed that the RNA levels of G3BP1 and G3BP2 were specifically reduced in depleted cells (Supplementary Figure 3A–3C). Of the 60,612 genes, 655 and 644 were upregulated, whereas 867 and 1,110 genes were downregulated in G3BP1- and G3BP2-depleted cells, respectively (Figure 6A, 6B). Out of these, 46.1–61.9% were mRNAs, while 16.5–29.7% were lncRNAs (Figure 6C–6F). We also validated RNA levels by RT-qPCR and confirmed upregulation of *neuroguidin* (*NGDN*) and *NADH:Ubiquinone oxidoreductase core subunit S2* (*NDUFS2*) and downregulation of *HMOX2* and *huntingtin* (*HTT*) RNA levels in G3BP1- and G3BP2-depleted NB cells, respectively (Supplementary Figure 3D–3F). Approximately 40% of mRNAs and lncRNAs upregulated by siG3BP1 and siG3BP2 overlapped, while 30–50% of those downregulated overlapped (Figure 6G, 6H). Most mRNAs whose expression levels were changed in either G3BP1- or G3BP2-depleted cells were abundant in SGs, and some possessed eCLIP-peaks for G3BP1 or G3BP2 (Figure 6I–6L). These results indicate that G3BP1 and G3BP2 directly bind to target transcripts for protein-coding genes and either positively or negatively regulate their expression. In contrast, more than half of the lncRNAs that changed in each depleted cell condition were enriched in SGs without eCLIP-peaks for G3BP1 or G3BP2 (Supplementary Figure 3G–3J), suggesting that the expression levels of lncRNAs were not regulated by direct interaction with G3BP proteins.

To understand how the assembly of SGs affects the transcriptome, we determined steady-state RNA levels in AS-treated SH-SY5Y cells and identified thousands of genes with altered RNA levels (Supplementary Figure 3K). Out of the upregulated genes, 34.1% were mRNAs, while only 14.9% of downregulated genes consisted of mRNAs (Supplementary Figure 3L, 3M). Out of the upregulated and downregulated genes, 28.4% and 30.8% were lncRNAs, respectively (Supplementary Figure 3L, 3M). Most mRNAs whose expression levels were changed under stress conditions accumulated in SGs, some with eCLIP-peaks for G3BP1 and G3BP2 (Supplementary Figure 3N, 3O). In contrast, 26.7% and 19.1% of lncRNAs whose expression levels were upregulated and downregulated, respectively, upon stress stimuli were enriched in SGs without G3BP1 and G3BP2 binding (Supplementary Figure 3P, 3Q). These results suggest that treatment with AS promotes the binding of G3BP1 and G3BP2 to hundreds of mRNAs, subsequently leading to their accumulation in SGs where their expression levels are regulated. Stress also elicits independent changes in the RNA levels of some lncRNAs within SGs in an independent manner of G3BP proteins. Notably, the number of mRNAs and lncRNAs whose expression levels were changed by treatment with AS was greater than those changed upon G3BP1- or G3BP2-depletion, and most of them hardly overlapped (Supplementary Figure 3R, 3S), suggesting that environmental and physiological stresses can affect RNA levels of rather broad genes, some of which are under the control of G3BP proteins.

We further explored the different distributions of major AD risk factors, including *APP*, *microtubule-associated protein tau* (*MAPT*) and *presenilin1* (*PSEN1*) [1, 2, 44, 45], in SGs, and found that *APP* and *ADAM metallopeptidase domain 10* (*ADAM10*) [46] were enriched in SGs and exhibited both G3BP1 and G3BP2 eCLIP-peaks (Supplementary Figure 3T). The expression level of *MAPT* was decreased upon depletion of *G3BP1* and *G3BP2* and treatment with AS, suggesting that G3BP proteins and SGs assembly are likely to prevent the *MAPT* transcript from mRNA degradation.

### SG-enriched RNA levels correlate with disease severity in human AD brains

To understand how SGs consequently contribute to the pathophysiological relevance of proteins in human AD brains, we explored the potential association between pathological changes in protein levels in AD brains and RNAs sequestered in SGs. To this end, we leveraged the dataset of a large-scale and deep multi-layered proteomic network analysis of AD brains from two sources: the Religious Orders Study and Memory and Aging Project (ROSMAP) and the Banner Sun Health Research Institute [47], in which over 1,000 brain tissues from control (*n* = 106), asymptomatic AD (AsymAD, *n* = 200), and AD (*n* = 182) brains were subjected to quantitative proteomics. A total of 8,619 proteins were used to build the protein co-expression network, which revealed 44 AD-correlated modules (M1–M44) [47], as listed in Supplementary Figure 4A. AsymAD cases exhibited neuropathologically comparable levels of Aβ plaques and tau tangles to AD cases but lacked significant cognitive impairment near the time of death, which is inferred to be an indicator of early preclinical stages of AD [48].

**Figure 7:**
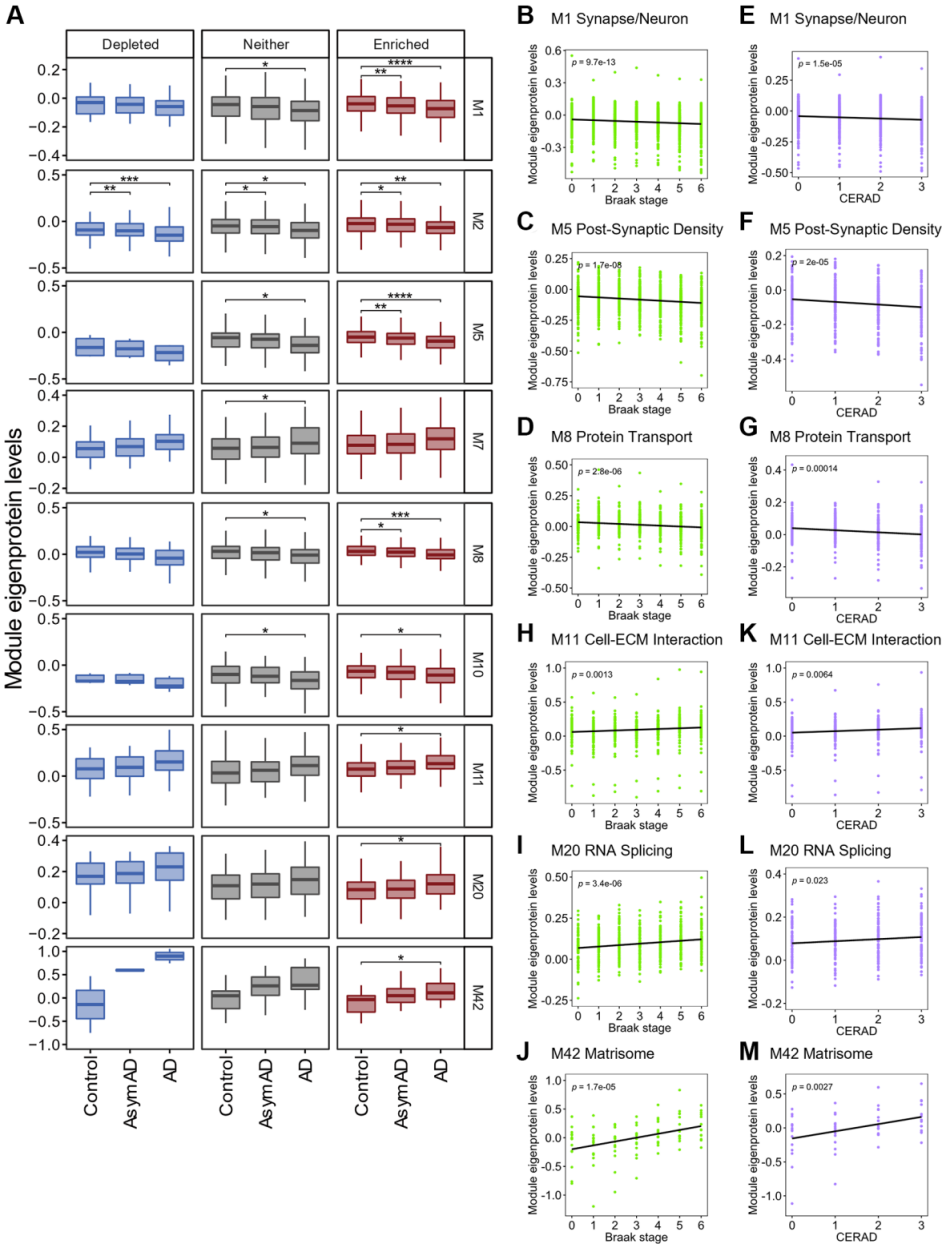
**Association between SG-enriched RNAs and changes in protein levels in AD brains.** (**A**) Box plot depicting Module eigenprotein levels by AD case status for the 9 modules statistically significant. Statistical significances were assessed by one-way ANOVA with Tukey test. No mark means not significant. ^*^q < 0.05, ^**^q < 0.01, ^***^q < 0.001, ^****^q < 0.0001. See also Supplementary Figure 4. (**B**–**M**) Plots showing Module eigenprotein levels of six SG-enriched RNA associated modules by BRAAK and CERAD scores. *p* indicates *p*-value for Pearson correlation coefficient.

We classified proteins in each module into three groups according to the distinct distribution of RNA groups to SGs, namely SG-enriched, depleted, and “Neither” RNAs, and then compared changes in protein levels across control, AsymAD and AD cases within each module. Out of 132 sets (44 modules × 3 SG RNA groups), 21 showed a significant difference in protein levels (Supplementary Figure 4B), corresponding to nine modules (Figure 7A). Protein levels in the M2 module were significantly decreased in AD and AsymAD across all three RNA groups. The M7 module showed a significant increase in protein levels in AD cases only in the “Neither” group. The M10 module also showed a significant decrease in protein levels in AD cases in the SG-enriched group and “Neither” group. Therefore, SGs are less likely to regulate protein levels in these three modules during the onset of AD. Notably, the remaining six modules, M1 Synapse/Neuron, M5 Post-Synaptic Density, M8 Protein Transport, M11 Cell-extracellular matrix (ECM) Interaction, M20 RNA Splicing, and M42 Matrisome, showed significant changes in protein levels in AD patients relative to the controls more clearly in the SG-enriched group. In particular, the M1, M5, and M8 modules showed significant decreases even in AsymAD cases compared to controls. Protein levels were negatively correlated with two distinct neuropathological scores: the Braak (Figure 7B–7D) and the Consortium to Establish a Registry for Alzheimer’s Disease (CERAD) scores (Figure 7E–7G), suggesting that RNAs enriched in SGs in these modules might cause pathophysiological processes in the early stages of AD before cognitive decline. In contrast, protein levels in the M11, M20, and M42 modules were significantly increased only in the SG-enriched group in AD cases relative to control. Positive correlations were observed with Braak (Figure 7H–7J) and CERAD scores (Figure 7K–7M), suggesting that RNAs in these modules were accumulated in SGs, which further led to progress in AD pathogenesis.

The principal biology of each module was originally determined by Gene Ontology (GO) analysis [47], where closely related biological processes increased in each module, even for SG-enriched genes (Supplementary Figure 4C). Notably, despite the M42 module being strongly enriched in ECM-associated proteins, including ECM- and glycosaminoglycan-binding proteins [47, 49], SG-enriched genes in the M42 module were enriched in genes with GO terms related to the development of neuronal cell networks such as axon guidance, neuron projection guidance, and axonogenesis. To identify genes that integrally control these biological processes, we performed Gene-Concept Network analysis using SG-enriched AD-associated genes in six modules (Supplementary Figure 4D). We examined the top 12 nodes that were the most significantly enriched GO terms, which apparently formed seven super nodes due to their close relationship and gene overlap. The analysis revealed genes exhibiting mutual interlinks across nodes, including a major AD risk factor APP, implicating that the disruption of these gene regulatory networks elicited by sequestration of transcripts into SGs may subsequently cause synaptic dysfunction, impairment of synaptic cytoskeletons, and learning/memory deficits in the brain, leading to AD development.

## DISCUSSION

In this study, we demonstrated using eCLIP-seq that both G3BP1 and G3BP2 proteins directly bind to numerous mRNAs, particularly to their 3′UTRs, and lncRNAs, and play a central role in their accumulation in SGs under stress conditions. The presence of lncRNAs within SGs indicated that prior translation is not a prerequisite for RNA accumulation in SGs. In principle, longer and AT-rich mRNAs are localized to SGs more efficiently, probably because more binding sites were provided for multiple RBPs [37]. In contrast, no such feature was observed for lncRNAs, suggesting that length may not play a role in targeting them to SGs. However, recognition by specific SG-resident RBPs may be required due to the large number of G3BP-binding sites SG-enriched lncRNAs possess. Additionally, most mRNA levels changed after the depletion of G3BP1 and G3BP2, and they exhibited direct RNA binding. However, in the case of lncRNAs, no such binding was observed, suggesting that G3BP proteins act to transport lncRNAs to SGs but are less directly associated with eliciting changes in lncRNAs levels. Notably, we demonstrated distinct changes in the RNA binding levels of G3BP1 and G3BP2 after stress. G3BP1 and G3BP2 exhibit different LLPS properties; G3BP2 is more prone to phase separation *in vivo* than G3BP1, likely due to its longer C-terminus composed of IDRs and an RRM, which conformationally switches upon stress [10], suggesting that these distinct molecular properties of G3BP proteins may be associated with the rearrangement of G3BP-bound RNAs before and after stress stimuli.

Importantly, we also showed that transcripts of AD-associated genes innately possess physical features (e.g., longer transcripts) which are likely to be sequestered by SGs and that they were included in SG-enriched RNAs, implicating SGs in the regulation of these genes. Furthermore, examination of the association between SG-enriched RNAs and the changes in protein levels in human AD brains revealed that six protein co-expression modules showed significant correlations with SG-enriched ones (Supplementary Figure 5), suggesting a link between RNA sequestration into SGs and impaired protein homeostasis in AD brains.

Three out of the six protein co-expression modules, M1 Synapse/Neuron, M5 Post-Synaptic Density, and M8 Protein Transport, showed significant decreases in protein levels according to disease severity [47]. Given that the translation of mRNAs within SGs is arrested, the sequestering of these mRNAs is likely to be associated with impaired protein synthesis and subsequent decline in protein levels. The acetylcholine receptor cholinergic receptor muscarinic 1 (CHRM1) belongs to the SG-enriched M1 module. In a mouse model of AD, the lack of *Chrm1* increased amyloidogenic processing of APP and exacerbated cognitive deficits [50]. In addition, reticulon 4 (RTN4), an M8 module member, interferes with BACE1 activity through direct interaction, thereby reducing Aβ levels [51]. Taken together, the decline in these protein levels mediated by SGs might cause AD development.

M11 Cell-ECM Interaction, M20 RNA Splicing, and M42 Matrisome modules are highly elevated in AD brains [47]. The M20 module with the SG-enriched group consisted of many RBPs, such as transcription and pre-mRNA processing factors, suggesting that persistent sequestration of these mRNAs to SGs could disrupt RNA metabolism in neuronal cells [8, 13, 27]. Notably, DNA methyltransferase 1 (DNMT1), an M20 module member, is the most abundant DNMT that maintains genomic methylation patterns [52]. In general, DNA hypermethylation is associated with the repression of gene expression by inducing heterochromatin formation [53]. Since DNA hypermethylation levels have been reported to be globally altered in human AD brains, such as the hypermethylated *apolipoprotein E4* (*APOE4*) promoter in late-onset AD patients [54], DNMT1-induced changes in DNA methylation levels, mediated by SGs, may affect AD onset and progression. The M11 and M42 modules were functionally close in the extracellular cell matrix. Importantly, the M42 module was enriched in genetic AD risk factors such as APP, which was enriched in SGs. Additionally, secreted frizzled-related protein 1 (SFRP1), a SG-enriched M42 module member, dampens ADAM10 α-secretase activity, thereby promoting amyloidogenic APP processing and Aβ production [55]. Another ADAM family member, ADAM17, also known as tumor necrosis factor-α-converting enzyme (TACE), belongs to the M11 module and primarily processes inflammatory cytokines, such as TNF-α, which trigger neuronal inflammation in the human brain, causing the loss of neuronal cells [56, 57]. Our findings suggest that SGs indirectly affect the increased protein levels in the M11 and M42 modules in AD development.

Examination of Gene-Concept Network analysis further revealed key regulatory gene networks that potentially link SG-regulating RNAs and pathological changes in protein levels in human AD brains. The prominent genes interlinked with the most GO term nodes were *APP* and *protein tyrosine kinase 2 beta (PTK2B)*. *PTK2B* is genetic AD risk factors that encode Ca^2+^-dependent protein kinases, PYK2 [58]. In a mouse model, Pyk2 has been shown to mediate Aβ-induced synaptic loss and cognitive deficits [59]. Several studies have suggested that intracellular Ca^2+^ homeostasis is dysregulated in human AD brains [60], which may lead to malfunction of Ca^2+^-dependent protein kinases, including PYK2. Sequestration of *PTK2B* transcripts to SGs and Ca^2+^ dyshomeostasis may synergistically affect synaptic loss, cognitive deficits induced by Aβ, and AD development.

These stresses provoke the formation of SGs, wherein a number of RNA molecules, including mRNAs and lncRNAs, are sequestered, which have detrimental roles in AD, probably leading to both enhanced and dampened protein levels in AD-associated protein co-expression modules. Determining the mechanism underlying RNA sequestration in SGs and the consequent changes in protein levels in AD brains will likely require further experiments in appropriate human neuronal models, animal models, and human clinical trials, which could represent a key goal in the discovery and development of suitable AD biomarkers and therapies.

## MATERIALS AND METHODS

### Cell culture and treatments

SH-SY5Y cells (ATCC, CRL-2266) were grown with 5% CO_2_ at 37°C in culture medium prepared from Advanced DMEM/F-12 (Thermo Fisher Scientific) supplemented with 10% fetal bovine serum (FBS), 2 mM L-Glutamine (Nacalai), and 1 × Penicillin-Streptomycin (Thermo Fisher Scientific). NB9 (RIKEN, RCB0477) and NB69 (RIKEN, RCB0480) cells were kindly gifted from Dr. Kyoko Fujiwara in Nihon University. NB9 cells were grown with 5% CO_2_ at 37°C in culture medium prepared from RPMI-1640 with 2 mM L-Glutamine (Nacalai) supplemented with 15% fetal bovine serum (FBS) and 1 × Penicillin-Streptomycin (Thermo Fisher Scientific). NB69 cells were grown with 5% CO_2_ at 37°C in culture medium prepared from RPMI-1640 (Nacalai) supplemented with 15% fetal bovine serum (FBS), 4 mM L-Glutamine (Nacalai), and 1 × Penicillin-Streptomycin (Thermo Fisher Scientific). For sodium arsenite (AS) exposure, the cell culture medium was replaced with fresh medium one hour prior to stress. Cells were then treated with 500 μM AS (Merck Sigma-Aldrich) for 1 h in 5% CO_2_ at 37°C. For gene knockdown, 5 × 10^5^ SH-SY5Y cells were incubated with 10 nM siRNA and 7.5 μL Lipofectamine RNAiMAX Reagent (Thermo Fisher Scientific) for 24 h with 5% CO_2_ at 37°C. After replacement with fresh medium, cells were incubated for an additional 48 h. Silencer Select siRNAs, s19754 (siG3BP1-1), siG3BP2 (Supplementary Table 1), and negative Control siRNA (siCtrl) were used.

### Enhanced cross-linking and immunoprecipitation (eCLIP)-seq

eCLIP-seq was performed as previously described [61] with the following modifications. A total of 1 × 10^7^ SH-SY5Y cells were used for each eCLIP experiment. For UV crosslinking, SH-SY5Y cells were washed once with ice-cold PBS. UV crosslinking was performed at an irradiance of 300 mJ/cm^2^ at 254 nm using a FUNA UV Crosslinker FS-1500 (Funakoshi). Cells were pelleted and lysed with lysis buffer containing 20 mM HEPES-KOH (pH 7.3), 150 mM NaCl, 1 mM EDTA, 1 mM dithiothreitol (DTT), 0.5% NP-40, and 1 × Protease Inhibitor Cocktail EDTA free (Nacalai). RNA–protein complexes were immunoprecipitated using 15 μg of antibody and 75 μL of Dynabeads Protein G magnetic beads (Thermo Fisher Scientific) for 2 h at 4°C. Beads were washed three times with wash buffer containing 20 mM HEPES-KOH (pH 7.3), 300 mM NaCl, 1 mM DTT, 0.05% NP-40, and 1 × Protease Inhibitor Cocktail EDTA free (Nacalai), followed by partial RNA digestion with 1 U/μL RNase T1 (Thermo Fisher Scientific) for 10 min at 37°C. Beads were washed three times with high-salt wash buffer containing 20 mM HEPES-KOH (pH7.3), 500 mM NaCl, 1 mM DTT, 0.05% NP-40, and 1 × Protease Inhibitor Cocktail EDTA free (Nacalai). For the visualization of protein-associated RNAs, the RNAs were dephosphorylated at their 5′ ends with Quick CIP (NEB) and then radioactively labelled with (γ-32P) ATP (PerkinElmer, NEG502A) and T4 polynucleotide kinase (T4 PNK, Thermo Fisher Scientific) on beads. The samples were analyzed by CosmoPAGE Bis-Tris gel electrophoresis (Nacalai) and visualized using Typhoon FLA 7000 (GE Healthcare). Immunoprecipitated proteins were visualized by silver staining using the Silver Stain 2 Kit wako (FUJIFILM Wako), according to the manufacturer’s instructions. For the construction of RNA-seq libraries, after 3-′ end RNA dephosphorylation with Quick CIP (NEB) and T4 PNK (Thermo Fisher Scientific) on beads, the RNAs were ligated at their 3′ ends to a barcoded 3′ RNA adapter with T4 RNA ligase 1 (NEB). Size-matched input (SMInput) controls were prepared for background. The samples were then subjected to CosmoPAGE Bis-Tris gel electrophoresis (Nacalai) and transferred to nitrocellulose membranes (FUJIFILM Wako). After cutting out the region of nitrocellulose containing RNA–protein complexes, RNAs were removed from the membrane by Proteinase K (Takara Bio) digestion. The isolated RNAs were reverse-transcribed using SuperScript IV (Thermo Fisher Scientific). The 5′ linker was ligated to the 3′ end of the cDNA fragments using T4 RNA ligase 1 (NEB). The adapters and linker used are shown in Supplementary Table 1. Libraries were then amplified using Q5 High-Fidelity DNA Polymerase (NEB) and size-selected via polyacrylamide gel electrophoresis. eCLIP libraries were sequenced using the HiSeq X platform with 150-bp paired-end reads in Macrogen.

### eCLIP-seq data analysis

Data processing was performed as previously described [61]. Adaptor sequences and low-quality reads were removed using fastp (version 0.23.2) [62]. Unique molecular identifiers (UMIs) were deduplicated using fastp. The reads were aligned to the human genome assembly GRCh38.p13 using STAR (version 2.7.10a) with default parameters. Peak calling was performed using CLIPper (version 2.1.2) with default parameters, and then normalized to remove background signals using SMInput. All eCLIP-seq experiments were performed using duplicates, and common peaks were used for analyses. The gene annotation file (Release 41 for GRCh38.p13) was downloaded from GENCODE [34]. Motif analysis was performed using the MEME suite XSTREME (https://meme-suite.org/meme/tools/xstreme) using the peaks.

### RNA immunoprecipitation (RIP)

Cells were lysed with lysis buffer containing 30 mM HEPES-KOH (pH 7.3), 150 mM KOAc, 5 mM MgOAc, 0.1% NP-40, 1 mM EDTA, and 1 × Protease Inhibitor Cocktail EDTA free (Nacalai). A total of 1 × 10^7^ cells were used for each experiment. RNA–protein complexes were immunoprecipitated using 5 μg of antibody and 25 μL of Dynabeads Protein G magnetic beads (Thermo Fisher Scientific) for 2 h at 4°C. Beads were washed five times with lysis buffer. RNAs were isolated using ISOGEN II (Nippon Gene) and RNA Clean and Concentrator-5 (ZYMO RESEARCH), and then reverse-transcribed to cDNA using the PrimeScript RT reagent Kit (Takara Bio) according to the manufacturer’s instructions. qPCR was subsequently carried out using KAPA SYBR FAST qPCR Master Mix (2X) Kits (KAPA BIOSYSTEMS) and QuantStudio 3 real-time PCR system (Thermo Fisher Scientific). The primers used are listed in Supplementary Table 1.

### Stress granule (SG) RNA-seq

SG RNA-seq was performed as previously described [37] with the following modifications. A total of 1 × 10^7^ SH-SY5Y cells were used for each experiment. After stress with AS, cells were washed once with ice-cold PBS and suspended in SG lysis buffer containing 50 mM Tris-HCl pH 7.4, 100 mM KOAc, 2 mM MgOAc, 0.5 mM DTT, 50 μg/mL heparin, 0.5% NP-40, 1 × Protease Inhibitor Cocktail EDTA free (Nacalai), and 0.05 U/μL RNase Inhibitor (TOYOBO), and then passed through a 25-gauge 5/8 needle attached to a 1 mL syringe 7 times, following centrifugation at 1,000 × g for 5 min at 4°C. The supernatant was collected in a new microcentrifuge tube and centrifuged at 1,000 × *g* for 5 min at 4°C. After centrifugation, 5% of the supernatant was collected to isolate total RNAs using ISOGEN II (Nippon Gene), following the manufacturer’s instructions. The remaining supernatant was processed to isolate the SG RNAs. To purify the SG cores, the samples were incubated with anti-G3BP1 antibody (BD Biosciences, 611126) for 1 h at 4°C in SG lysis buffer, followed by centrifugation at 1,000 × *g* for 5 min at 4°C. The pellet was resuspended in SG lysis buffer and incubated with Dynabeads Protein G magnetic beads (Thermo Fisher Scientific) for 3 h at 4°C in SG lysis buffer. After washing and Proteinase K (Takara Bio) digestion as previously described [37], SG RNAs were isolated using ISOGEN II (Nippon Gene) according to the manufacturer’s instructions. RNA-seq libraries were prepared and sequenced using the DNBSEQ platform as 150-bp paired-end reads in BGI. For validation of RNA levels in SGs, isolated RNAs were reverse-transcribed to cDNA using the PrimeScript RT reagent Kit (Takara Bio) according to the manufacturer’s instructions. qPCR was subsequently carried out using KAPA SYBR FAST qPCR Master Mix (2X) Kits (KAPA BIOSYSTEMS) and QuantStudio 3 real-time PCR system (Thermo Fisher Scientific). The primers used are listed in Supplementary Table 1.

### KD and AS RNA-seq

Total RNAs were isolated using ISOGEN II (Nippon Gene), following the manufacturer’s instructions. Poly-A RNA-seq libraries for G3BP1-depleted and G3BP2-depleted SH-SY5Y cells were prepared and sequenced using the DNBSEQ platform as 150-bp paired-end reads in BGI. RNA-depletion RNA-seq libraries for SH-SY5Y cells treated with AS were prepared and sequenced using the DNBSEQ-G400 platform as 150-bp paired-end reads in GENEWIZ. For validation of RNA levels, isolated RNAs were reverse-transcribed to cDNA using the PrimeScript RT reagent Kit (Takara Bio) according to the manufacturer’s instructions. qPCR was subsequently carried out using KAPA SYBR FAST qPCR Master Mix (2X) Kits (KAPA BIOSYSTEMS) and QuantStudio 3 real-time PCR system (Thermo Fisher Scientific). The primers used are listed in Supplementary Table 1.

### KD, AS, and SG RNA-seq data analysis

For KD and SG RNA-seq, adaptor sequences, contamination, and low-quality reads were removed from BGI. For RNA-seq of AS-treated cells, adaptor sequences and low-quality reads were removed using fastp (version 0.23.0) [62]. The reads were aligned to GRCh38.p13 using HISAT2 (version 2.1.0) with default parameters. The SAM files obtained were converted to BAM format using Samtools (version 1.10) [63]. Transcripts per million (TPM) was calculated using StringTie (version 2.1.6). The gene annotation file (Release 41 for GRCh38.p13) was downloaded from GENCODE [34]. All length data for the 5′UTR, ORF, 3′UTR, and total and GC content data were acquired using the Ensembl BioMart tool [64].

### Plasmid constructions and expressions

To yield the G3BP1-AcGFP expression plasmid, full-length *G3BP1* cDNAs and AcGFP sequences were amplified by RT-PCR from the SH-SY5Y cDNA library and by PCR from pAcGFP1-N1 (Clontech), respectively, and then inserted into pcDNA3 by NEBuilder HiFi Assembly (NEB). To yield the G3BP2-DsRed-Monomer (mDsRed) expression plasmid, the mDsRed sequence was amplified by PCR from pDsRed-Monomer-N1 (Clontech) and inserted into pcDNA3-G3BP2 by NEBuilder HiFi Assembly (NEB). The primers used are listed in Supplementary Table 1. Plasmid transfection and induction of protein expression in SH-SY5Y cells were performed using FuGENE HD (Promega), following the manufacturer’s instructions. Live cell imaging was performed using a FLUOVIEW FV10i (Olympus).

### Cell fractionation

SH-SY5Y cells were suspended in lysis buffer containing 20 mM HEPES-KOH (pH7.3), 150 mM NaCl, 1 mM EDTA, 1 mM DTT, 0.5% NP-40, and 1 × Protease Inhibitor Cocktail EDTA free (Nacalai). After centrifugation at 16,000 × *g* for 15 min at 4°C, the supernatant (Sup) was treated with trichloroacetic acid (TCA), precipitated to concentrated proteins and suspended in sodium dodecyl sulfate (SDS) sample buffer containing 50 mM Tris-HCl pH 6.8, 2% SDS, 10% glycerol, 100 mM DTT, and 0.02% bromophenol blue. The precipitate (Ppt) was lysed in the SDS sample buffer.

### Western blotting

SDS-PAGE gels were blotted onto PVDF membranes (Millipore, IPVH00010) using blotting buffer containing 192 mM glycine, 25 mM Tris, and 20% methanol. The blots were blocked using Blocking One (Nacalai). Primary antibodies to the following proteins were used: G3BP1 (ABclonal, A3968, 1:1000), G3BP2 (ABclonal, A6026, 1:1000) and actin (FUJIFILM Wako, 010-27841, 1:1000), which were also listed in Supplementary Table 1. HRP-Linked anti-Mouse IgG (Cytiva, NA931, 1:10000) and anti-Rabbit IgG (Cytiva, NA934, 1:5000) were used for secondary antibodies. The signals were detected using Pierce ECL Plus Substrate (Thermo Fisher Scientific). Images were captured and processed using a ChemiDoc Touch Imaging System (Bio-Rad).

### Quantification and statistical analysis

#### Statistical analysis and Gene Ontology (GO) analysis

We used the statistical packages implemented in R 4.2.0 for all calculations and plots in this study. Statistical tests are provided in the figures and figure legends. GO, Gene-Concept Network, and Kyoto Encyclopedia of Genes and Genomes (KEGG) pathway analyses were performed using the clusterProfiler package in R [65].

### Data and code availability

Deep sequencing datasets were deposited in the National Center for Biotechnology Information Gene Expression Omnibus (NCBI GEO) database and are available under the accession number GEO: GSE188397. No software was used for this project.

## Supplementary Materials

Supplementary Figures

Supplementary Table 1
