# Supplementary material for: Stress granules sequester Alzheimer’s disease-associated gene transcripts and regulate disease-related neuronal proteostasis
Source: Aging (Albany NY). 2023 May 22;15(10):3984–4011. doi: 10.18632/aging.204737 (PMC10258020; doi:10.18632/aging.204737)
Supplement: Supplementary Figures [file aging-15-204737-s001.pdf]

SUPPLEMENTARY FIGURES

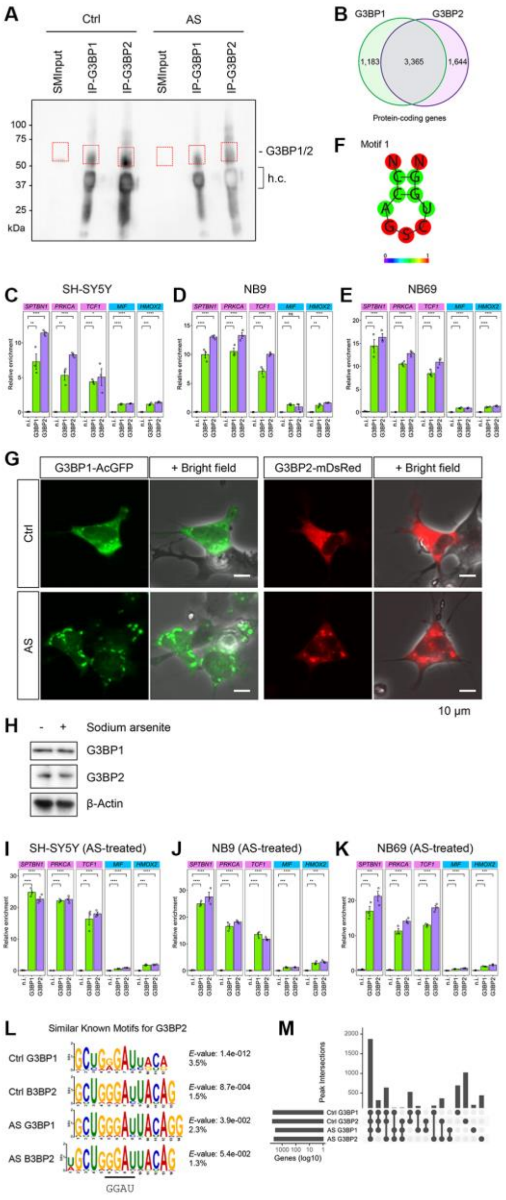

Supplementary Figure 1. Related to Figures 1 and 2. eCLIP-seq of 3BP1 and G3BP2 in SH-SY5Y cells. (**A**) Western blotting against immunoprecipitated (IP) G3BP1 and G3BP2 proteins before and after sodium arsenite (AS) treatment, confirming that both proteins were efficiently immunoprecipitated. SMInput is the size-matched input control. Red dotted squares indicate the gel-extracted regions for eCLIP-seq libraries. (**B**) Venn diagram depicting the overlapped mRNAs that possess G3BP1 and G3BP2 eCLIP-peaks. (**C**–**E**) Bar charts depicting the interaction between G3BP proteins and *SPTBN1*, *PRKCA*, *TCF4*, *MIF*, and *HMOX2* RNAs by RIP in SH-SY5Y (**C**), NB9 (**D**), and NB69 cells (**E**). n.i.: non-immune IgG used as an IP negative control. Error bars represent standard error (SE) from three independent experiments. Statistical significances were assessed by Student's *t*-test. ns: not significant, $^{*}p < 0.05$, $^{**}p < 0.01$, $^{***}p < 0.001$, $^{****}p < 0.0001$. (**F**) Predicted RNA structure for motif 1 by CentroidFold. The color bar represents the probability of a base pair. S and N indicate G/C and any ribonucleotides, respectively. (**G**) Live imaging showing the cellular localizations of G3BP1-AcGFP and G3BP2-mDsRed in SH-SY5Y before (Ctrl) and after (Arsenite) sodium arsenite treatment. (**H**) Western blotting of G3BP1 and G3BP2 before and after AS treatment, indicating that the protein levels of G3BP1 and G3BP2 were unchanged by AS. (**I**–**K**) Bar charts depicting the interaction between G3BP proteins and *SPTBN1*, *PRKCA*, *TCF4*, *MIF*, and *HMOX2* RNAs by RIP in SH-SY5Y (**I**), NB9 (**J**), and NB69 cells (**K**) under stress condition. n.i.: non-immune IgG used as an IP negative control. Error bars represent SE from three independent experiments. Statistical significances were assessed by Student's *t*-test. $^{**}p < 0.01$, $^{***}p < 0.001$, $^{****}p < 0.0001$. (**L**) Similar known motif for G3BP2 [36] enriched within eCLIP-peaks of G3BP1-and G3BP2-bound RNAs. (**M**) Intersection across mRNAs that possess G3BP1 and G3BP2 eCLIP-peaks.

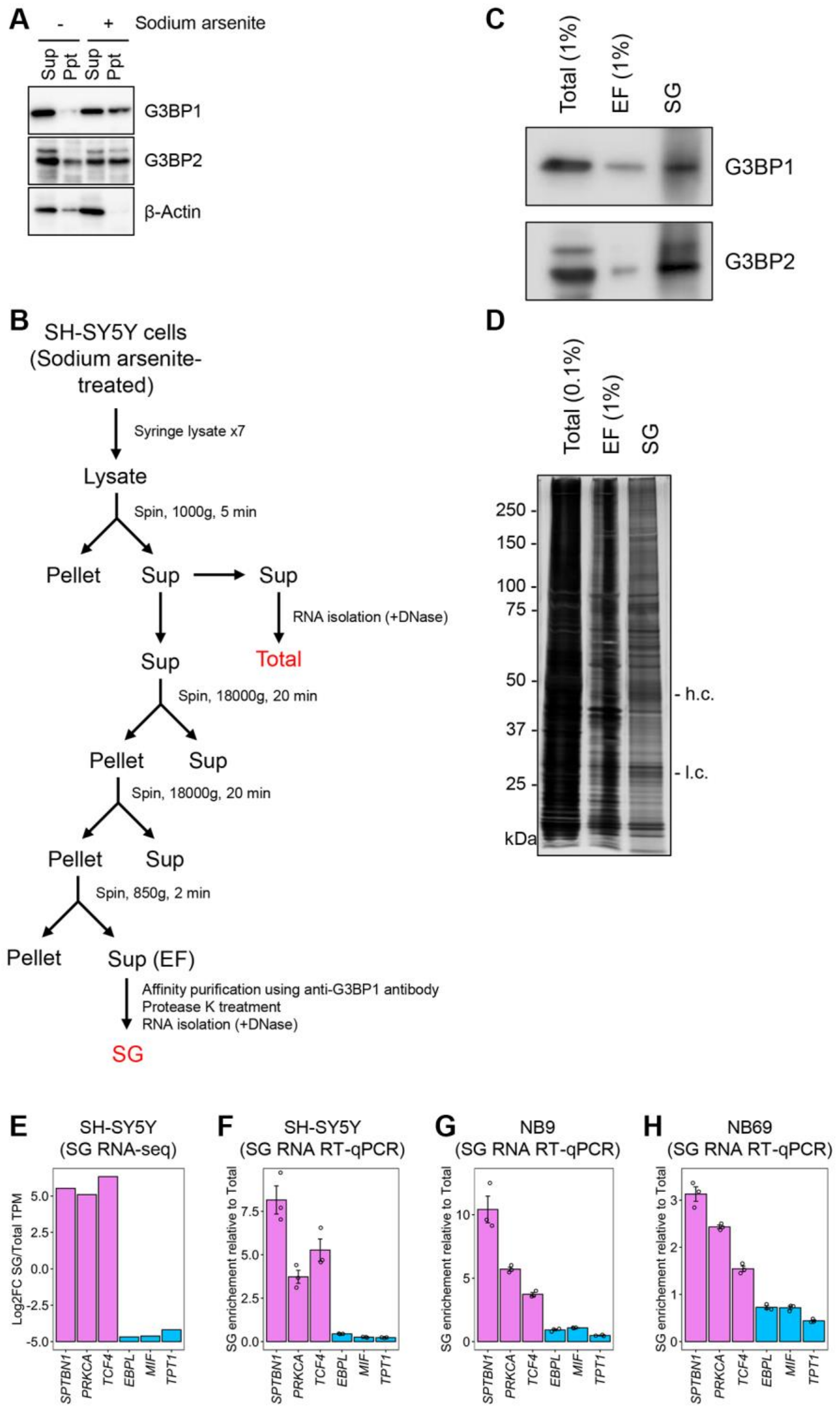

Supplementary Figure 2. Related to Figure 3. Isolation of SG cores in SH-SY5Y cells. (**A**) Western blotting of G3BP1 and G3BP2 following cellular fractionated SH-SY5Y cells before and after sodium arsenite treatment. Sup, supernatant; ppt, precipitate. Actin was used as a marker for cytoplasm. (**B**) Schematic representation of the SG purification process in SH-SY5Y cells. In brief, after SG formation induced by sodium arsenite, SH-SY5Y cells were subjected to syringe lysis, followed by a series of centrifugation steps. The SG cores were then affinity purified using anti-G3BP1 antibody and treated with proteinase K. RNA was isolated via ISOGEN II extraction, yielding purified SG core RNA. EF, SG-enriched fraction; SG, SG cores. (**C**) Western blotting of G3BP1 and G3BP2 following affinity purification of SG cores. (**D**) Silver staining following affinity purification of SG cores. (**E**) Bar charts depicting Log2-fold change of SG/Total TPM scores for the indicated genes in SG RNA-seq data. (**F**–**H**) Bar charts depicting the relative RNA levels of the indicated genes in SG cores determined by RT-qPCR in SH-SY5Y (**F**), NB9 (**G**), and NB69 cells (**H**). Error bars represent SE from three independent experiments.

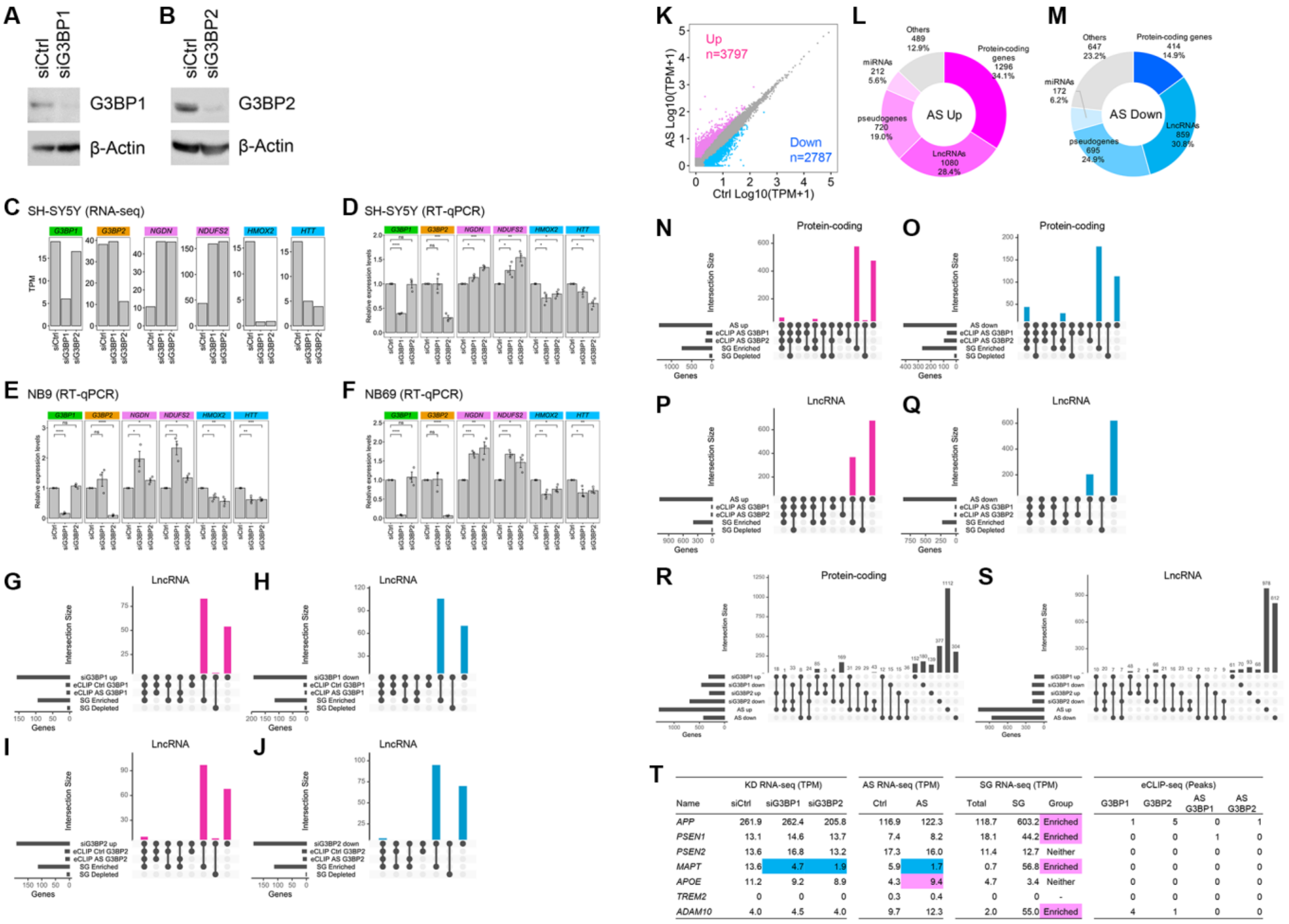

T

	KD RNA-seq (TPM)			AS RNA-seq (TPM)		SG RNA-seq (TPM)			eCLIP-seq (Peaks)			
Name	siCtrl	siG3BP1	siG3BP2	Ctrl	AS	Total	SG	Group	G3BP1	G3BP2	AS G3BP1	AS G3BP2
APP	261.9	262.4	205.8	116.9	122.3	118.7	603.2	Enriched	1	5	0	1
PSEN1	13.1	14.6	13.7	7.4	8.2	18.1	44.2	Enriched	0	0	1	0
PSEN2	13.6	16.8	13.2	17.3	16.0	11.4	12.7	Neither	0	0	0	0
MAPT	13.6	4.7	1.9	5.9	1.7	0.7	56.8	Enriched	0	0	0	0
APOE	11.2	9.2	8.9	4.3	9.4	4.7	3.4	Neither	0	0	0	0
TREM2	0	0	0	0.3	0.4	0	0	-	0	0	0	0
ADAM10	4.0	4.5	4.0	9.7	12.3	2.0	55.0	Enriched	4	1	0	0

Supplementary Figure 3. Related to Figure 6. Changes in RNA levels upon the depletion of G3BP1 and G3BP2, and AS treatment in SH-SY5Y cells. (**A**, **B**) Western blotting of G3BP1 (**A**) and G3BP2 (**B**) in G3BP1- and G3BP2-depleted SH-SY5Y cells, respectively. (**C**) TPM scores of G3BP1 and G3BP2 in control (siCtrl), G3BP1-depleted (siG3BP1), and G3BP2-depleted (siG3BP2) cells. (**D**–**F**) Bar charts depicting changes in RNA levels of the indicated genes determined by RT-qPCR in G3BP1 and G3BP2-depleted SH-SY5Y (**D**), NB9 (**E**), and NB69 cells (**F**). Relative expression levels were calculated by normalizing to *GAPDH*. Error bars represent SE from three independent experiments. Statistical significances were assessed by Student's *t*-test. ns: not significant, $^{*}p < 0.05$, $^{**}p < 0.01$, $^{***}p < 0.001$, $^{****}p < 0.0001$. (**G**–**J**) Intersections across upregulated, downregulated genes upon knockdown, and enriched RNAs based on eCLIP-seq and SG RNA-seq of lncRNAs. (**K**) Scatter plot depicting RNA abundance in AS-treated cells. (**L**, **M**) Pie charts depicting the relative contribution of gene categories for upregulated (**L**) and downregulated (**M**) genes upon AS-treatment. (**N**–**Q**) Intersections across upregulated and downregulated genes upon AS-treatment and enriched RNAs based on eCLIP-seq and SG RNA-seq of protein-coding genes and lncRNAs. (**R**, **S**) Intersections for upregulated or downregulated genes of protein-coding genes (**R**) and lncRNAs (**S**) upon knockdown and AS-treatment. (**T**) RNA levels of major AD risk factors upon depletion of G3BP1 and G3BP2, AS treatment, enrichment to SG, and G3BP1 and G3BP2 eCLIP-peaks. Red and blue backgrounds indicate upregulated and downregulated genes, respectively. SG-enriched genes were also indicated with red background.

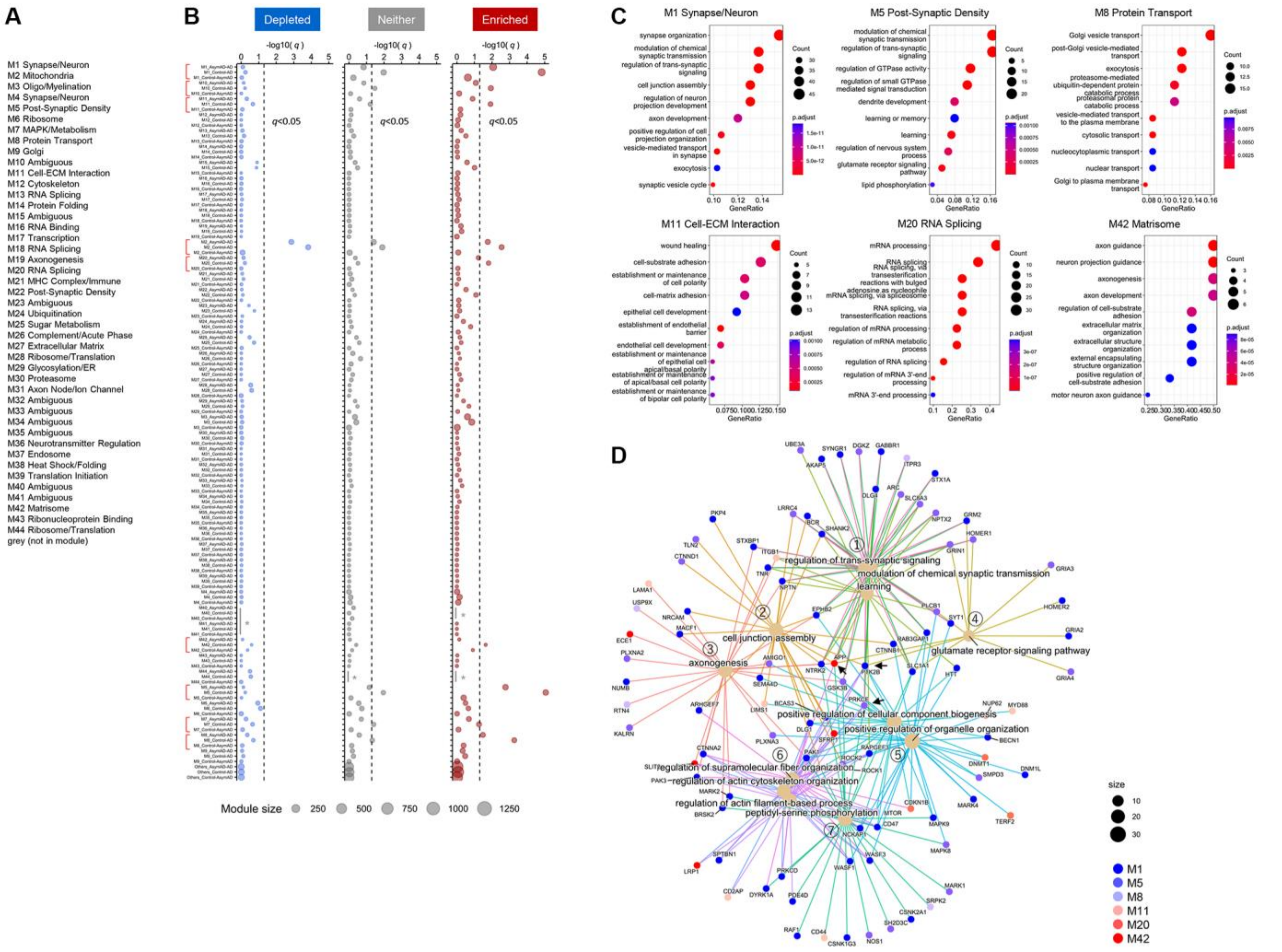

Supplementary Figure 4. Related to Figure 7. Association between SG-enriched RNAs and changes in protein levels in AD brains. (**A**) List of 44 protein co-expression modules modified from Johnson et al. [47]. (**B**) Correlation between SG-enriched RNA levels and Module eigenprotein levels. Differences of protein expression level by case status in each module were assessed by one-way ANOVA with Tukey test and shown as -log10 (q-value). The dashed lines indicate q = 0.05, above which enrichment was considered significant. The red square brackets indicate nine modules containing sets that showed significant changes in protein levels in SG-enriched, depleted, or "Neither" groups. Asterisks (*) indicate no proteins included. (**C**) GO analysis for SG-enriched genes classified in each module. (**D**) Gene-Concept Network analysis for SG-enriched AD-associated genes classified in six modules.

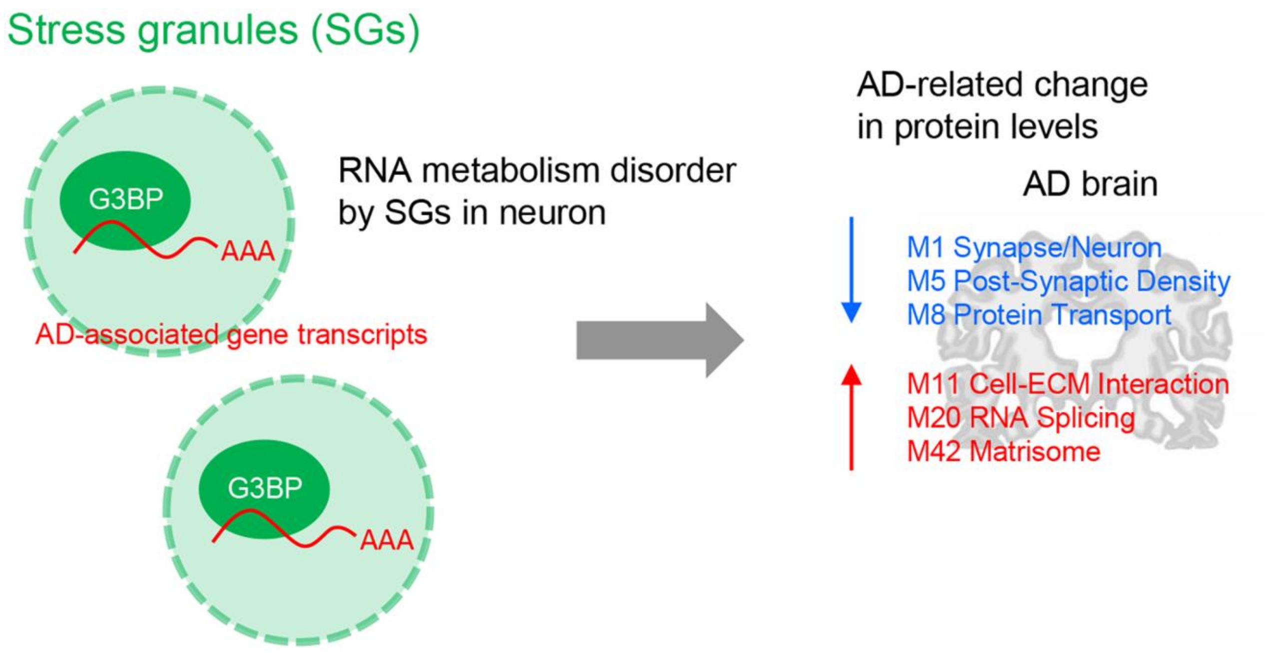

Supplementary Figure 5. A model for the mechanism of sequestration of AD-associated gene transcripts by SGs and its impact on disease-related proteomic changes. SGs sequester RNAs, which consequently cause changes in protein levels of six modules associated with the development of AD.
