# Supplementary material for: Stress granules sequester Alzheimer’s disease-associated gene transcripts and regulate disease-related neuronal proteostasis
Source: Aging (Albany NY). 2023 May 22;15(10):3984–4011. doi: 10.18632/aging.204737 (PMC10258020; doi:10.18632/aging.204737)
Supplement: Supplementary Table 1 [file aging-15-204737-s002.pdf]

SUPPLEMENTARY TABLE

Supplementary Table 1. List of experimental resources.

Primers

Name	Sequence (5′ to 3′)	Experiment
pcDNA3-Flag-F2	ACAAGTGAGATATCCATCACACTGGCG	PCR for pcDNA3-G3BP1-AcGFP
pcDNA3-Flag-R1	TCCATCACGAATTCGCCCTTGTCATCA	PCR for pcDNA3-G3BP1-AcGFP
G3BP1-F3	CGAATTCGTGATGGAGAAGCCTAGTC	PCR for full-length G3BP1 cDNA
G3BP1-R4	CGGTGGATCCTGCCGTGGCGCAAGC	PCR for full-length G3BP1 cDNA
AcGFP-F1	CGGCAGGATCCACCGGTCATGGT	PCR for AcGFP cDNA
AcGFP-R1	GGATATCTCACTTGTACAGCTCATCCA	PCR for AcGFP cDNA
mDsR-F2	GTCGCCTCGAGGACAACACCGAGGACGT	PCR for mDsRed cDNA
mDsR-R2	GCCCTCTAGACTACTGGGAGCCGGAGT	PCR for mDsRed cDNA
pFG3BP2-F1	AGTAGTCTAGAGGGCCCTATTCTATAG	PCR for pcDNA3-G3BP2-mDsRed
pFG3BP2-R1	TGTCCTCGAGGCGACGCTGTCCTGTGA	PCR for pcDNA3-G3BP2-mDsRed
GAPDH-qPCR-F	CTAGCCTCCCGGGTTTCTCT	qPCR for GAPDH
GAPDH-qPCR-R	TCGACAGTCAGCCGCATCT	qPCR for GAPDH
G3BP1-qPCR-F	CTTGGGCATCTGTGACCAGT	qPCR for G3BP1
G3BP1-qPCR-R	GGGCTGTGAAGCTGGTACTT	qPCR for G3BP1
G3BP2-qPCR-F	TGTGGAACTTCGCATCAATACC	qPCR for G3BP2
G3BP2-qPCR-R	AAACGTACTTCCCCTCGAAACA	qPCR for G3BP2
SPTBN1-qPCR-F1	CGGTGGACACAAGCGAAATG	qPCR for SPTBN1
SPTBN1-qPCR-R1	TACGATCAGAGGTCGGGGAG	qPCR for SPTBN1
PRKCA-qPCR-F1	AGAGGGACGTGAGAGAGCAT	qPCR for PRKCA
PRKCA-qPCR-R1	CCTTTGCCACACACTTTGGG	qPCR for PRKCA
TCF4-qPCR-F1	TGGGGGTTAAGGAGAAGTGTTT	qPCR for TCF4
TCF4-qPCR-R1	TCCAAGTTGCCACATTGCTTC	qPCR for TCF4
EBPL-qPCR-F1	GTGGAAATTCTGACCGTCGC	qPCR for EBPL
EBPL-qPCR-R1	TCTGCAGGAAATGCCGGTAA	qPCR for EBPL
MIF-qPCR-F1	GAACCGCTCCTACAGCAAGC	qPCR for MIF
MIF-qPCR-R1	GGCCGCGTTCATGTCGTAAT	qPCR for MIF
TPT1-qPCR-F1	TCAGCCACGATGAGATGTTCT	qPCR for TPT1
TPT1-qPCR-R1	TCCTACTGACCATCTTCCCCT	qPCR for TPT1
NGDN-qPCR-F1	GAGAAAGGACGGCGAAAACG	qPCR for NGDN
NGDN-qPCR-R1	AGTTCCCCCTGTCAAAGCAC	qPCR for NGDN
NDUFS2-qPCR-F1	CCCAAGCAAAGAAACAGCCC	qPCR for NDUFS2
NDUFS2-qPCR-R1	GGGCCCAAAGTTCAGGGTAA	qPCR for NDUFS2
HMOX2-qPCR-F1	CAGCGGAAGTGGAAACCTCA	qPCR for HMOX2
HMOX2-qPCR-R1	TCCGAGAGGTCAGCCATTCT	qPCR for HMOX2
HTT-qPCR-F1	CTCGTCAGCTTGGTTCCCAT	qPCR for HTT
HTT-qPCR-R1	AGAGATTTGGGAGCACTGGC	qPCR for HTT

siRNAs

Name	Sense (5′ to 3′)	Antisense (5′ to 3′)	Note
siG3BP2	CCGUAGAAUAAUUCGCUAUCC	AUAGCGAAUUAUUCUACGGUU	Silencer Select siRNA

eCLIP-seq adapters

Name	Sequence (5′ to 3′)	Note
RNA_X1A	/5Phos/AUAUAGGNNNNNAGAUCGGAAGAGCGUCGUGUAG/3SpC3/	3′ adapter for IP
RNA_X1B	/5Phos/AAUAGCANNNNNAGAUCGGAAGAGCGUCGUGUAG/3SpC3/	3′ adapter for IP

RiL19	/5phos/AGAUCGGAAGAGCGUCGUG/3SpC3/	3′ adapter for SMInput
AR17	ACACGACGCTCTTCCGA	Reverse transcription
rand103Tr3	/5Phos/NNNNNNNNNNAGATCGGAAGAGCACACGTCTG/3SpC3/	5′ linker

Antibodies

Name	Experiment	Source	Identifier
Anti-G3BP1	eCLIP, SG RNA	BD Biosciences	611126
Anti-G3BP1	Western blotting	ABclonal	A3968
Anti-G3BP2	eCLIP	abcam	ab86135
Anti-G3BP2	Western blotting	ABclonal	A6026
Anti-Actin	Western blotting	FUJIFILM Wako	010-27841
Non-immune IgG (n.i.)	Negative control	IBL	17314

Software and algorithms

Name	Version	URL
STAR	2.7.10a	https://github.com/alexdobin/STAR
HISAT2	2.1.0	http://daehwankimlab.github.io/hisat2/
Samtools	1.10	http://www.htslib.org/
fastp	0.23.2	https://github.com/OpenGene/fastp
StringTie	2.1.6	https://ccb.jhu.edu/software/stringtie/
CLIPper	2.1.2	https://github.com/YeoLab/clipper
R	4.2.0	https://www.r-project.org/
RStudio	Build 461	https://www.rstudio.com
